# Zootherapeutic uses of animals excreta: the case of elephant dung and urine use in Sayaboury province, Laos

**DOI:** 10.1186/s13002-021-00484-7

**Published:** 2021-10-28

**Authors:** Jean-Marc Dubost, Phommachack Kongchack, Eric Deharo, Palamy Sysay, Chithdavone Her, Lamxay Vichith, Duffillot Sébastien, Sabrina Krief

**Affiliations:** 1 Museum National d’Histoire Naturelle-UMR 7206, Paris, France; 2 Department of Pharmaceutical Sciences, Faculty of Pharmacy, University of Health Sciences, Vientiane, Lao PDR; 3 Department of Botany, Faculty of Natural Sciences, National University of Laos, Vientiane, Lao PDR; 4 Elephant Conservation Center, Nam Tien Reservoir, Xayabury District, Lao PDR; 5 UMR 152 Pharmadev, IRD, UPS, 35 chemin des maraîchers, Université Paul Sabatier, 31062 Toulouse, France; 6 Food and Drug Department, Ministry of Health, Vientiane, Lao PDR

**Keywords:** Zootherapy, Elephas maximus, Asian elephant, Lao PDR, Feces, Faeces, Urine, Zoonotic transmission

## Abstract

**Background:**

Despite a widespread aversion towards faeces and urine, animal excreta are used in traditional medicine in many countries since centuries, but records are scattered and few therapeutic uses have been accurately documented while in the current context of emerging zoonoses such records may be of major interest.

**Methodology:**

In this study, we investigated the therapeutic uses that mahouts in Xayaboury province, Lao PDR make of elephant urine and faeces as well as of the brood chamber that beetles (*Heliocopris dominus*) fashion from elephant dung. Semi-structured interviews were conducted with mahouts on elephant diet, health problems and responses to disease, andwhether they use elephant products. Data were supplemented by interviews with traditional healers.

**Results:**

Seven respondents reported the use of elephant urine in ethnoveterinary care for elephants and in human medicine in case of diabetes and otitis. 25 respondents reported therapeutic use of elephant faeces (EF) and elephant dung beetle brood chambers. The major indications are gastrointestinal and skin problems. Macerations or decoctions are drunk or used externally as a lotion. The mahouts attribute the therapeutic effectiveness of EFs to their content which includes the remains of many species from the elephant diet which they consider to be medicinal.

**Discussion:**

The indications of these uses are consistent with pharmacological and clinical studies highlighting the properties of different animals’ urine and faeces and their curative potential tested in vivo. The acknowledgement by the mahouts of medicinal properties of elephant faecal bolus contrasts with the rare justifications of animal material use recorded in zootherapeutic studies, which falls within the symbolic domain. However, numerous studies highlight the preponderant role of the microbiota in physiological processes, raising the hypothesis of a curative action of EF, by rebalancing the user’s microbiota.

**Conclusion:**

The therapeutic uses of EF preparations despite their possible curative properties are a potential source of zoonotic transmission from elephants to humans. In the current context of globalisation of trade which favours the emergence of zoonoses and in relation with the issue of One Health, it becomes crucial to further document the zootherapeutic practices to prevent emerging diseases. As elephants and local related ethnoethological knowledge are threatened, documenting them is urgent to contribute to their preservation.

**Supplementary Information:**

The online version contains supplementary material available at 10.1186/s13002-021-00484-7.

## Background

### Zootherapy and uses of animal excreta

Zootherapy, in the sense of traditional therapeutic uses of substances from the animal world emerged in the 1990s as a particular field of ethnomedicine and ethnopharmacology studies. Zootherapic practices involve the use of all kinds of animal matters including different animal body parts, secretions (blood, milk, venom, bile, musk, cocoons, spiderwebs), some animal productions (such as honey, propolis, nests) and excreta (urine, faeces, ambergris, kidney stones). Although these latter, and especially animal urine and faeces on which this study focuses, are often the subject of learnt or adaptive aversion [1–3], medicinal uses of animal excreta are actually recorded in the oldest treatises of the learned medical traditions that have come down to us: as in the ancient Egyptian [4], Ayurvedic [5, 6], Chinese [7, 8], Tibetan [9, 10], Greek [11, 12] and Hebrew [13] traditions, and remained in common use in Western scholarly medicine at least until the Enlightenment, as shown in a book written in the eighteenth century by the German doctor Franz Christian Paullini, whose title in English would be “How most diseases and damages could be fortunately cured by excrement and urine” [14].

**Fig. 1:**
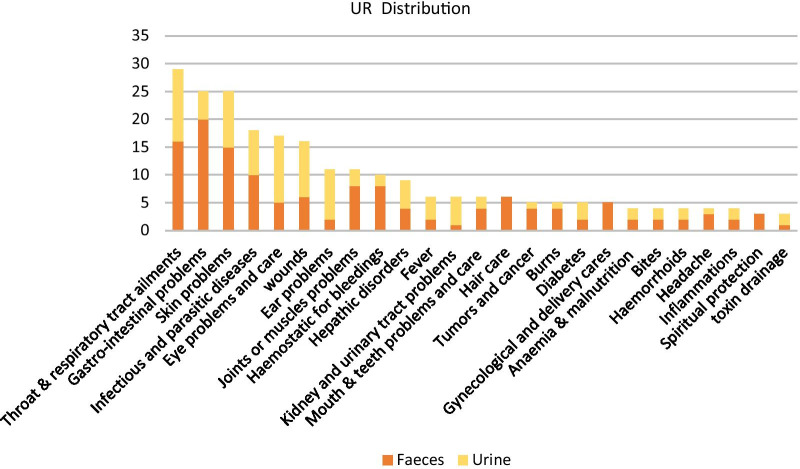
Distribution of UR by category of indications

Regarding recent or contemporary traditional uses of these matter we found 220 use reports (UR) recorded in 64 zootherapic studies of which 129 relate to faeces and 91 to urine. These UR are compiled in Additional file 1: URs of animals excreta. The animal species whose urine or faeces are used are distributed in the very diverse zoological classes of insects, reptiles, birds, and wild and domestic mammals. Among the uses of mammal excreta, 30 UR of human urine and 6 UR of human faeces are reported.^1^ The indications of these zootherapic uses are very diverse, as shown on Fig. 1

This great variety of indications is to be compared with the wide diversity of metabolites and microbiota composing the faeces of the different animal species used, or for urine with the wide spectrum of indications that its antimicrobial and emollient properties can cover (see below).

In these URs, urine is used as it is or mixed with mud, crushed plant material, flour or rice to form a paste. It is drunk, sometimes sweetened or diluted in water or applied locally, either as a lotion or as a poultice when used in a paste, can be instilled in the eyes or ears, or used as an enema. Faeces are used fresh, dried in the sun, roasted then pulverized, burned and reduced to ashes, in decoction or maceration and sometimes combined with other ingredients such as plant parts, honey, curd, figs and flour (baked into bread for hepatitis). In one UR, the stomach and intestine of the animal (porcupine) are dried with their contents and consumed. The methods of administration are also very varied: in internal use they can be ingested as they are (pigeon faeces), drunk in the form of a decoction or maceration, simply sniffed or sometimes the smoke from burning faeces is inhaled; in external use, they are applied locally, sometimes as a hot poultice, inserted into the nose (haemostatic) or used to massage the affected area.

This brief overview of therapeutic uses of animal faeces and urine shows that these practices date back to the known origins of human medicine, and have been and are still widely distributed.

The motivations or the *modus operandi* on which the uses of animal materials identified are based is poorly documented in the surveys that present them. If we did not find justifications for the use of animal urine or faeces in the zootherapy studies consulted, a few studies report motivations underlying the use of other animals matter: Alves et Alves [16] reports that in Brazil “reptiles that move slowly are used to calm people”; Pemberton [17], recording the use of animal materials by traditional doctors in South Korea quotes the example of centipedes “with their numerous legs, feet and articulated body segments [which] are used for leg, foot and joint problems”; Friants et al. [18] report that in Senegal heads and whole bodies of Rüppel’s Horseshoe bat *(Rhinolophus fumigatus)* were used for the treatment of mental illness because "bats exist as a symbol of orientation, and therefore could aid patients who […] lacked mental orientation”, and in Nigeria the use of dog saliva to cure dog bites or snake teeth to cure snake bites, both uses that the authors interpret as an illustration of the ‘like cures like’ theory of healing [18]. All these examples are given by the authors who quote them to illustrate quite similar analogic rationales: one establishing a link via an analogy between the physical or behavioural characteristics of the animal whose products are used and the symptoms of the ailment considered—rationale that Pemberton [17] compares with the European doctrine of signatures that have been developed and formulated as such in Europe during the sixteenth and seventeenth centuries [7]—and the other which, as is the case in homeopathy, consist in the use of the agent that causes a disorder to treat that same disorder. It is not excluded though, as Bennett points out in his discussion of the theory of signatures, that these “signatures” may be sometimes a posteriori rationalisations to account for an observed effective therapeutic action of the substance used, constituting moreover an excellent mean of memorising and transmitting these uses.^2^

### Environmental issues

The use of animals and their products for therapeutic purposes raises the question of the pressure on the environment and the threats to biodiversity posed by these practices, a problem that is all the more acute with the globalization of international trade, which encourages a demand for “exotic” therapeutic products. With regard to the materials of interest here, although a priori the use of animal faeces or urine does not imply the capture of individuals of the species considered, in some cases their collection and preparation does involves the killing of the animal. As for a species of flying squirrel (*Biswamoyopterus biswasi*) classified as critically endangered by the IUCN from which the bladder is removed to collect its urine which is used in case of kidney or gallstones by an ethnic group in north-east India [19], or for a use of porcupine (*Histrix indica*) in northern India whose stomach and intestine are consumed with their content.

### Elephants in Laos and uses of elephant excreta

In the course of a survey carried out in Laos, taking the bond and proximity of life that unites mahouts and elephants to study how observation of animals may have contributed to the development of human pharmacopoeia [20], zootherapic uses emerged among other medicinal practices, extending the scope of the survey to the question of the origin of the zootherapic practices that we examine here. The elephant is an emblematic species in Laos, formerly called Lan Xang or the Kingdom of a Million Elephants. Although enjoying an eminent status in this country, whose foundation is legendarily associated with this animal [21, 22], the wild elephant (*Elephas maximus*) population of Laos which was estimated at 2000–3000 in 1988 [23] had drastically reduced to just 600–800 individuals by 2009 [24]. Domestic elephant numbers are also declining sharply and are now comparable to the number of their wild counterparts [24, 25]. These elephants living in contact with humans have an intermediate status between wild and captive or domesticated animals. Traditionally sourced from wild populations and most elephant calf births in the village being the result of females mating with wild males in the forest [26, 27], they have not undergone a selection process like most domesticated animals, and in Laos their population constitutes a genetic pool as important as that of wild elephants.

According to traditional management methods in the area studied, village elephants are not fed and are still periodically released into the forest for long periods [20, 28, 29]. However, beside this traditional way of living the relation with elephants, the globalisation, industrialisation and the increased use of western medicine are modifying both the relationship and the local medicinal practices. Laos is currently witnessing a transfer of village elephants—which are no longer used for transporting goods and are less and less employed in the now regulated logging industry—to elephant resorts where young inexperienced mahouts (elephant caretaker) are often employed [29], leading to a disruption in the transmission of traditional knowledge related to elephants. In our previous study mentioned above [20], we have highlighted a set of medicinal and ethnoveterinary practices that stem from this proximity of life that unites mahouts and their elephants and the use that both species make of the resource of their environment. This valuable knowledge is threatened by the reduction of the forest cover which shelters the resource used and by the relocation of village elephants. It is therefore urgent to document this knowledge, which is part of the cultural heritage of Laos and which could contribute to better management of the health and well-being of the elephants in tourist resorts.

We present here a study of zootherapy practices that are also rooted in this close relationship between mahouts and their elephants, allowing us to better understand the motivations that may have led to their emergence. These practices consist in the therapeutic use of elephant urine and faeces including the fecal material found in the brood chambers that beetles fashion from the elephant dung. We will place these uses in perspective with the other similar uses of animal excreta presented in Additional file 1: URs of animals excreta and discuss their therapeutic potential in the light of the mahouts’ own justifications for their therapeutic efficacy, pharmacological studies carried out on these substances, and the emerging uses of human faeces in biomedicine. Because zoonotic transmission of pathogens is a risk that has become particularly acute in the context of globalization and multiplication of trade, and the multiplication of zoonotic pandemics, we also aim to raise awareness of the risk of related to human practices involving the exchange of substances and biological material with animals. Thus, in this still little explored field of therapeutic uses of animal faeces, we expect that this study will shed light on traditional practices involving the handling, the topical application, and the ingestion by humans of a material containing a particularly developed microbial flora and therefore potentially contaminant.

## Material and methods

### Study site

**Fig. 2:**
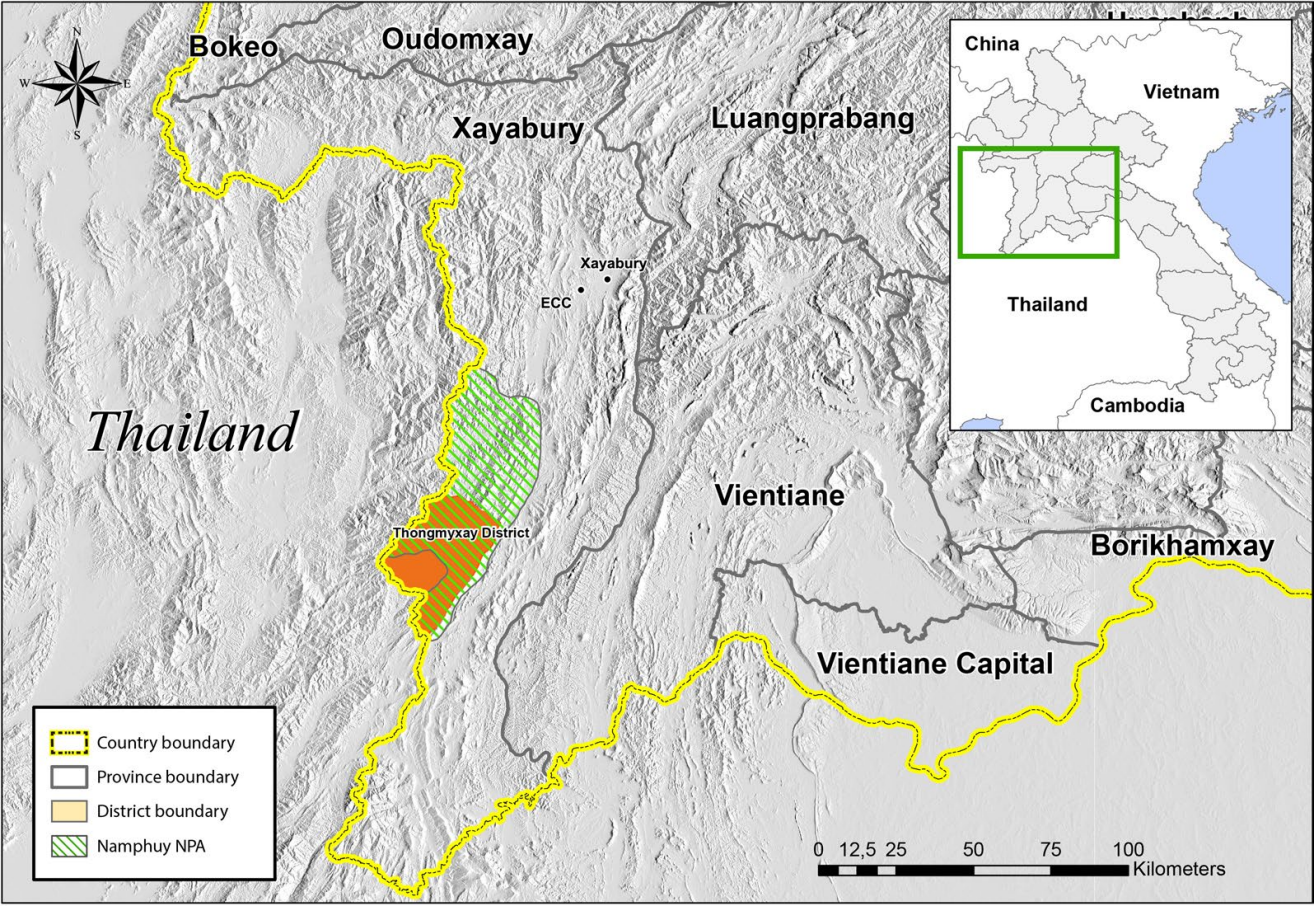
Contextual map of Thongmyxay district and location of the ECC centre *(Juan A. Torres)*

This study was conducted at two sites in Sayaboury province (Fig. 2), which is home to 80% of the village and captive elephants in Laos [30]:

1. The district of Thongmyxay, whose inhabited area is encompassed by the Namphuy National Protected Area (NNPA) covering 1912 km^2^ and shelters the second largest population of wild elephants in Laos, estimated at 60–80 individuals in 2009 [24]. The inhabited area is mainly surrounded by forested areas consisting of degraded secondary forests, *Dypterocarpaceae* forests, riverine forests and hill bamboo forests.
2. The Elephant Conservation Centre (ECC) in the north of the province, dedicated to elephant conservation and research in Laos, develops ecotourism activities focused on elephant observation and research programmes for both wild and captive elephants. It is delegated by the Ministry of Forests to be responsible for the NNPA’s wild elephant protection activities.

**Fig. 3:**
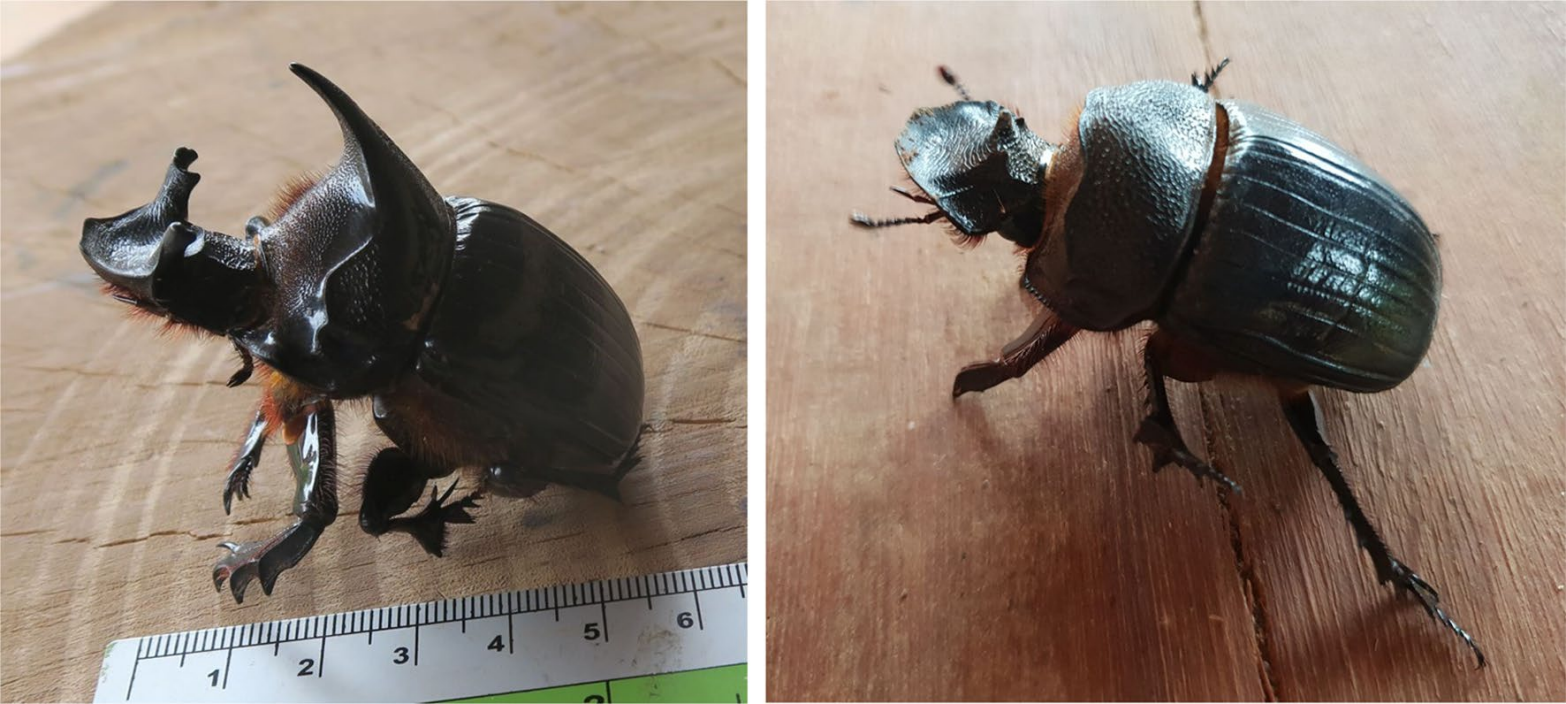
Heliocopris dominus male (left) and female (right) (photo JM Dubost)

**Fig. 4:**
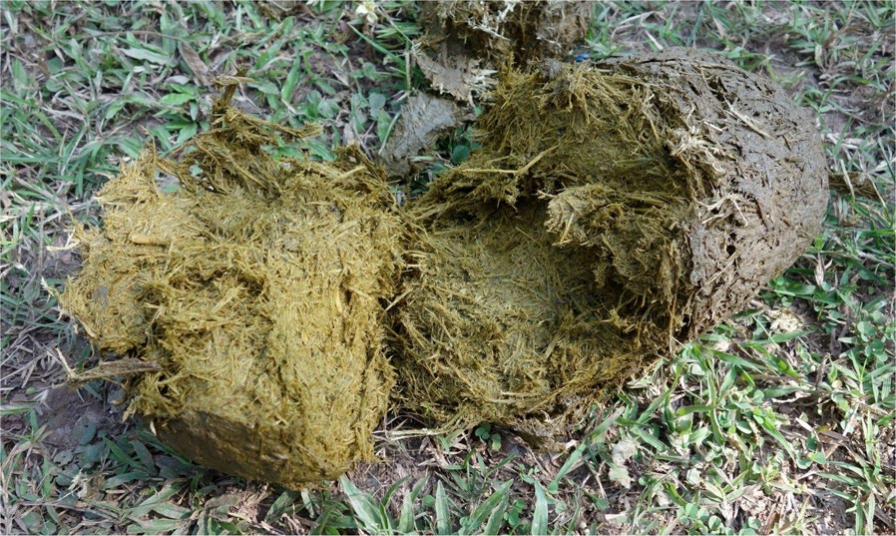
Open elephant dung (photo JM Dubost)

### Data collection

Sixty-six mahouts aged 35 to 78 were interviewed in the same province of Xayabury, 36 in Thongmyxay district and 29 at the ECC (Xayabury district), of which 13 were also from Thongmyxay, bringing the number of mahouts from Thongmyxay interviewed to 49, and the remainder were from other districts of Xayabury province (Xayabury district: 7, Piang: 4, Paklai: 3), except for one mahout who originated from Bokeo province. Fifty-four of the mahouts are from a mahout lineage and 40 are still active. All mahouts in Laos are male. Semi-structured individual interviews were conducted on elephant management practices and diet, health problems, their needs, the possible care provided to them, and observations regarding the response elephants give to their health problems. Mahouts were also asked whether they use or know about uses of elephant products (Figs. 3, 4).

We carried out an inventory of plants which the mahouts indicated were consumed by elephants, specifying the parts consumed. Four traditional healers (1 female and 3 males) were interviewed and asked which species from this inventory they used for therapeutic purposes. We supplemented these interviews with that of a woman whose father was a mahout and who was referred to us for her experience in the use of elephant dung beetle brood chambers.

## Results and discussion

### Uses of elephant urine

Seven mahouts out of the 66 interviewed reported therapeutic uses of elephant urine. Two of them use it in ethno-veterinary preparations to treat their elephant: they mix it with the chopped stem of *Alpinia galanga* (L.) Willd. One of them uses it in a pad in case of skin rash to apply lotion to the affected area, and the other adds dried elephant dung to the preparation that he applies with his hands on skin abrasions resulting from the chafing of chains. The other five mentions of therapeutic uses of urine are applied to humans, they are not the respondents’ own uses or a home practice, but uses that they have heard of and that they report. Four mahouts say that people have asked them for elephant urine for cases of diabetes. One of them specifies that they drink a little every day, but don’t know for how long, and that they say they are happy with it as it works well. Baird [31] reports a similar use in southern Laos: “In Phatthoumphone District, Champasak Province, diabetics boil 5 L pots of elephant urine and ingest the medicine over a number of days to treat their condition. Buckets are regularly placed under Phatthoumphone’s many domesticated elephants to collect the urine.” Two other similar urine uses for diabetes are mentioned in our bibliographical inventory (Additional file 1: URs of animals excreta): one in India where 5 ml of fresh urine of mainland Serow (*Capricornis sumatraensis*) is taken once a month, and one in Sikkhim where fresh urine of *Bos Taurus* diluted in some water is drunk [32, 33]. Another use of elephant urine given by one mahout consists of instilling elephant urine in the ear to treat ear infections. Nine similar uses are recorded in Additional file 1 where urine from different mammals is used for ear problems; these are dogs (*Canis lupus familiaris*), musk deer (*Maschus* sp.), barking deer (*Muntiakus munjak*)*, Bos Taurus indicus* and *bos indicus* in India, Tibetan argali (*Ovis ammon odgsoni*) in Nepal and aardvark *(Orycteropus afe)* in Nigeria [34–41]. When the method of administration is specified, it also consists of an instillation in the ear (8 UR).

### Uses of elephant faeces (EF) and elephant dung beetle brood chambers (EBC)

Twenty-nine mahouts (44% of the mahouts interviewed) reported the use of EF or EBC. Five of them mentioned prophylactic uses of EF to purify hen houses and keep insects and parasites away, either by placing fresh dung in the hen house, or by fumigation by burning dry dung. Twenty-five respondents reported personal or family therapeutic practices of EF or EBC: an elderly woman born into a mahout family in Thongmyxay, 23 mahouts from Thongmyxay, representing 47% of the 49 mahouts from this district interviewed, and one mahout from Hongsa district in the same province of Xayabury, whom we met and interviewed at ECC. We explain below why we address the uses of EF and EBC together. Uses recorded for each respondent are presented in the Table 1.

#### The elephant dung beetle

During our survey, while we were addressing the issue of human use of elephant products, fifteen mahouts and one woman whose father was a mahout, all fromThongmyxay, mentioned therapeutic uses of elephant dung beetle brood chambers (EBC). This scarab beetle has been identified as *Heliocopris dominus* from pictures of specimens brought by mahouts.

K. Joseph [42, 43] has studied this coprophagous species in India, which depends exclusively on the fresh dung of elephants. During the months of July and August, in the rainy season, the females dig a tunnel of 20 to 50 cm under the pile of elephant droppings they exploit, ending with a horizontal chamber. They transport 800 to 2,400 g of dung into this chamber,^3^ which they then shape into 2 or 4 balls of 9 to 12 cm in diameter that they cover with a thin layer of soil and in which they create a cavity that will house an egg. This egg hatches after a week, releasing a larva which, passing through three instar larva stages, develops over four months by feeding from the inside of its nest. This development period is followed by a three-month quiescent period, during the dry season, at the end of which the larva enters a pupal stage. The adult insect emerges from this pupa at the end of the dry season and feeds on the remains of the nest until the first monsoon rains, which soften the soil sufficiently to allow it to dig its way out.

In the zootherapy studies consulted we found only one mention of therapeutic use of beetle nests (*sp.* Not specified) reported in an inventory carried out in a rural municipality in Brazil[44]. These nests are used for the treatment of mumps, but the method of preparation or administration is not indicated.

#### Therapeutic uses of EF or EBC

The major indications for EF and EBC are gastrointestinal problems (17 UR: diarrhoea, stomach-ache, food poisoning accompanied by vomiting) and skin problems (10 UR: skin rashes, inflammations, and fungal infections).

Other indications mentioned were diabetes, fevers, and the condition of ‘internal heat’ (*hon nay*) described as a feeling of warmth in the abdomen, distinct from the rise in temperature called fever in biomedicine. Two ethno-veterinary uses were reported for the treatment of elephant wounds and sores. A mahout who is also a *mo phi* (traditional healer using supernatural means for healing) and sometimes also uses medicinal preparations, makes use of elephant faeces to treat problems related to neuromotor problems sometimes affecting the mobility of the limbs: faeces wrapped in a piece of cloth are placed in a stream for 15 to 20 min to form a pad used to massage and dab the affected limbs. A mahout finally reports having heard that in the south of Laos children are given a decoction of EF to drink so that they become “as strong as an elephant”, but he specified that in Thongmyxay he has never heard that people do so. We have also heard about such uses for children in the South of Thailand. Table 2 summarizes these uses by category of indications with the methods of administration used.

**Table 1:** Use reports of EF and EBC (one row for each respondent)

EF/EBC	Indication	Preparation and use
EF	Ethnoveterinary use for elephants’ skin abrasion	Urine and dry elephant faeces are mixed with crushed *Alpinia galanga* stems (in equal amounts) and applied by hand on the wound
EF	Ethnoveterinary use for elephants’ wounds	A poultice made of a mixture of clay and elephant faeces in equal parts placed in a piece of banana stem is applied on wound
EF	Diabetes, diarrhoea, intoxication with vomiting	Decoction of sun-dried faeces drunk at will
EF	Fever	Maceration of dry faeces, collected fresh or dry, 1 handful per litre drunk all day
EF	Fever, diarrhoea, digestive problems, skin rash	Decoction drunk in case of diarrhoea or fever, or used as a lotion for rash. Alcoholic maceration in case of digestive problems
EF	Neuromotor problems as an outcome of stroke (‘sen pasat’)	Faeces wrapped in cloth are placed in a stream for 15–20 min and used to massage and dab the limbs of affected people. Female faeces are used to treat males and vice versa
EF	Food intoxication with vomiting	Decoction of dried faeces (one handful per litre of water, boiled for 10 min) drunk all day long
EF	Diarrhoea, food intoxication	Decoction of dried droppings
EF	Skin rash	Aqueous maceration of dry faeces used in lotion
EF	Diarrhoea, skin rash	Decoction drunk for diarrhoea or used as a lotion for skin rash
EBC	Food intoxication (e.g. from toxic mushroom ingestion)	Aqueous maceration drunk at will until recovery
EBC	Diarrhoea, food intoxication	Decoction drunk. 1/3 of EBC used for each preparation
EBC	Skin rash	Decoction drunk
EBC	Tinea pedis (athlete’s foot)	Pat the affected area with a decoction twice a day
EBC	Diarrhoea, food intoxication	Decoction drunk 3 times a day. Sometimes wood and bark of *Broussonetia papyrifera* and culm of *Dendrocalamus brandisii* are added to the decoction
EBC	Skin rash	Decoction used in lotion
EBC	Diarrhoea	Decoction drunk for 2–3 days (one fifth of EBC with ¾ litre of water sometimes with sugar cane added)
EBC	Diarrhoea, stomach-ache	Decoction drunk
EBC	Diarrhoea, food intoxication	Decoction with roots of *Strobilanthes auriculatus* Nees (one thumb of each for 1 L of water) drunk
EBC	Food intoxication, stomach-ache	Decoction or aqueous maceration drunk
EBC	Stomach-ache	Decoction drunk (one handful for 3 L of water to be drunk at discretion until recovery)
EBC	Diarrhoea, stomach-ache, internal heat (‘hon nay’), skin problems	Diarrhoea, stomach-ache, internal heat: decoction drunk (2 thumbs of EBC for l litre of water). Skin problems: decoction used in lotion
EBC	Skin irritation	Decoction drunk or used in lotion
EBC	Diarrhoea, fever, skin rash	Diarrhoea, fever: decoction drunk. Skin rash: decoction drunk or used in lotion. Use ½ EBC for 4–5 L of water
EBC	Stomach-ache, bloating	Decoction of EBC with plants: drunk (1 L per day)

**Table 2:** UR compiled by indication categories and matter used

	Indications		
	Gastro-intestinal problems	Skin problems	Other
Faeces (10 r.)	Decoction drunk (5) Alcoholic maceration drunk (1)	Maceration used in lotion (1) Decoction used in lotion (2)	-Diabetes: decoction drunk (1) -Ethnoveterinary use for wounds in a poultice applied topically (2) -Fever: decoction (1) or maceration drunk [1] -Nerve (*sen pasat*) and neuromotor problems (1)
EBC (15 r.)	Decoction drunk (9) Maceration drunk (2)	Decoction (7): drunk (3) used in lotion (5)	Internal heat (‘*hon nay*’) decoction drunk (1)
Total of UR	17 UR	10 UR	7 UR

Number of UR are given in brackets for each type of use

Two mahouts use EBC in combination with plants to prepare a decoction against diarrhoea: in once case the roots of *Strobilanthes auriculatus* Nees, (an Acanthaceae also used for the same indication by one of the local healers interviewed), and for the other, the bark of *Pentace burmanica* Kurz, seeds of ‘*naiken mak kiao*’ (a palm tree not collected) and a sp. of Artemisia (cf. *Artemisia indica* Willd) used whole with the root. In the two ethnoveterinarian uses noted, elephant faeces are applied as a poultice to treat wounds or sores affecting elephants. For all uses, dry or freshly collected faeces are used, but then dried beforehand in the sun. EBC are used when collected or kept in a dry place. Two mahouts specify that it is preferable to use bull elephant faeces to treat females and cow elephant faeces to treat males. For macerations or decoctions, about a handful of dung is used per litre of water, which is taken for internal use at discretion throughout the day, or two to three times a day. For EBCs, we have noted the following proportions which are more variable according to the respondents: one thumb for 1 L of water, ½ EBC for 4–5 L of water, one handful for 3 L of water.

Two mahouts had simply heard of therapeutic uses of EF, but had not directly witnessed their use. One of them reported that at the Xayabury Elephant Festival (a popular annual festival attended by the local population and Lao and foreign tourists) some people asked him for fresh dung to treat themselves, but he did not know the intended use. For the 25 respondents who reported personal or family therapeutic practices, one of them pointed out that these products are no longer used now that they can get ready-made medicines, while others reported recent use of them. It is difficult to estimate the actual current prevalence of these practices because they take place in the general context of a decline of traditional medicinal practices in favour of the use of biomedicine; one of the mahouts explained that medicinal plants used to be planted around the house to have them at hand in case of need, but that now people go to the district dispensary for healthcare needs, these species are tending to disappear from the neighbourhood. He added that as the number of village elephants decreases, it becomes more difficult to find elephant dung beetle tunnels. This assertion is in line with the findings of a study carried out in Malaysia on beetles visiting elephant dung which states that most of the species encountered are not found in locations where elephants are absent [45]. Thus another mahout, 48 years old, mentioned EBC use as a family custom that was more common in his childhood. Since respondents were not explicitly asked when the last occurrence of the uses they reported occurred, it is possible that some people who reported therapeutic use of elephant dung may have been referring to past use that they still consider to have the potential for future use in case of need.

In other countries, elephant dung is reported to be used in Nigeria against epilepsy, to heal wounds, for swelling or bone problems, in the case of cancer, fever, dysentery, pile, haemorrhage, and headache [18], in Tanzania against children’s convulsions [46], in Zimbabwe to ease labour and delivery for women [47, 48], in Ethiopia against headache [49], in Angola for rheumatism [50] and in India for skin infections and to ease teething [51]. See Additional file 1: URs of animals excreta for all these UR. Regarding the use of animal faeces for indications similar to those for which Thongmyxay’s mahouts use EF and EBC we noted in the same inventory 12 UR for dermatological problems (cf. Additional file 1): the use of faeces from different bovids in India (*Bos indicus* and *Bos bubalus*) [52–55], in Pakistan (*Bos primigenius indicus*) [56], in Laos (black cow *sp.* not specified) [31] and in South Africa (*Bos Taurus*); for other animals, dove faeces (*Columba livia, C. palomas, C. oenas*) in Spain [41], in India faeces of crow (*Corvus splendens*) [57]; in Pakistan, the faeces of Nightingale (*Luscinia megarhynchos*) are used[58] and in Sudan the faeces of one humped camel (*Camelus dromedarius*)[59]; in Brazil the faeces of hens (*Gallus domesticus*) are used in ethnoveterinary care [60]. For these dermatological uses, the mode of administration when specified consists of a topical application. For gastrointestinal disorders, 19 uses of faeces are reported: 10 in India: camel faeces (*Camelus dromedarius*) [53, 55, 57], dhole faeces (*Canis alpinus*) [61], donkey faeces (*Equus asinus*) in ethnoveterinary use [62], house sparrow faeces (*Passer domesticus*) [39, 63, 64], Porcupine (*Hystrix indica*) [64] and human faeces –to induce vomiting- [36]; two in Nigeria: African elephant *(Loxodonta cyclotis)* and Palm civet *(Nandinia binotata),* two in Sudan: Crested porcupine (*Hystrix cristata*) and Hedgehog (*Hemiechinus aethiopicus*) faeces [59]; two in Spain: dog faeces (*Canis lupus familiaris*) [65]; one in South Africa cow (*Bos taurus*) faeces[66]; one in Portugal: house mouse faeces (*Mus musculus*) [67], one in the Philippines: goat faeces [68]. With the exceptions of one use of dromedary faeces where these are burned and their ashes applied to the belly and the use of house sparrow faeces which are applied to the anus of constipated babies, in the other uses a maceration or decoction of the faeces is prepared and administered internally through the oral route except for one UR where a maceration of Palm civet *(Nandinia binotata)* faeces is used as an enema for babies’ stomach pains [18]. Against fever, two uses are reported in Nigeria: in one UR African elephant *(Loxodonta cyclotis)* faeces are mixed with Gin and drunk [18], and in the other one, cow faeces *(Bos taurus)* are used but the mode of use is not specified [66]. Against diabetes, one use of cow faeces *(Bos taurus)* is reported in Nigeria and one use of grasshoppers (*sp.* not specified) faeces in Sudan [59].

### Justifications given by mahouts for the use of elephant excreta

#### Elephant Urine

The seven mahouts who mentioned therapeutic uses of elephant urine did not give an explanation of their potential therapeutic efficacy. It should be noted that these mahouts reported uses that they had heard of but did not personally practice.

#### Elephant Faeces and EBC

##### Explicit reasons

Out of the 25 respondents who reported therapeutic uses of EF or EBC, seven put forward some explanations to account for the therapeutic efficacy of these matters. They are all related to the elephant’s diet and the care it takes in selecting what it consumes, and were given for both the direct use of EF (3 r.) and EBC (4 r.): three mahouts told us that these substances are good for humans because elephants eat a lot of good plants; four others pointed out that among these plants are medicinal plants, which are sometimes difficult to find (1 r). One mahout emphasizes the diversity in the elephant’s diet by stating that it is preferable to use wild elephant faeces because they have a more diverse diet than their domestic counterparts. This assertion should also be linked to the fact that in Laos food from the forest is especially prized for its special virtues [50]. One of the mahouts made the link between these elements, saying that the elephant knows how to cure itself and how to choose the right plants for this purpose, and that this is why the elephant dung beetle brood chambers made with their faeces are used. This also shows clearly that what is sought in the EBC is primarily the material contained in the elephant faeces within it and not the beetle brood chamber itself, that is why we have addressed the uses of EF and EBC together in the previous section. Thus, although two respondents told us that elephant dung beetle larvae, or the insect when it emerges from the pupal stage and is still tender as well as the larvae of another dung beetle called *chu chi kwan*—buffalo dung beetle (*sp.* not identified)—which makes its nest with buffalo dung—are a delicacy, no mahout mentioned any use of buffalo dung beetle nests. When asked why EBC is used rather than EF, respondents generally had no explanation, saying that this had always been the practice in the family. One person, however, explained that EBC are preferred to EF because the insect fragments the faeces into small pieces, which makes the preparation more efficient. This remark may be linked to that of another mahout who says that he prefers to use the faeces of young elephants to the one of adults because they chew their food carefully. These assertions makes good sense if one conceives these operations as facilitating the availability of curative plant substances that their faeces may contain.

##### Implicit elements

Awareness that elephants cure themselves with plants is widely shared among mahouts. Twenty-six mahouts reported practices of preferential consumption of plant items by females during their breeding period, or by their elephants when they are sick, behaviours that they interpret as a form of self-medication [20]. In addition, mahouts inspect their elephant’s faeces, which provides information about the animal’s health *(ibid)*, so it is a matter they are familiar with and they know the texture and high content of partially digested plant debris. When asked if elephant calves consume anything other than their mother’s milk, 36 mahouts told us that their mother’s dung is the first solid element they consume. Four mahouts point out that since elephant calves cannot yet grasp plants well and have no teeth, their mother’s faeces are a food that is easier for them to access and assimilate. The same allocoprophagic behaviour has been documented in foals and young zebras [69–71], elephant calves (*Elephas maximus*) [72, 73], and some hatchlings [74], all of which consume the faeces of their mothers or close relatives. Elephant calf allocoprophagy is also generally well known to keepers of domesticated elephants in Asia, as shown in this recommendation for the care of orphaned elephants at weaning time given in the “Elephant Care Manual for Mahouts and Camp Managers” [72]: “you must find dung from a mature elephant and that dung must be fresh and from a fit, healthy elephant […and] offer a lump about the size of a fist to the calf every day for a month or two”.

Various hypotheses have been put forward to explain these behaviours in juvenile animals from different species and their possible or proven benefits. These hypotheses involve semiochemical interactions with the mother, nutritional supplementation and, finally, maturation of their digestive microbiota by the addition of intraspecific microbial material, the latter hypothesis being the most widely shared [69, 71, 73–75]. An interesting interpretation of this elephant calves behaviour was given to us in a study conducted in southern Thailand in 2014 [76], by two brothers who had an elephant that they left to forage in the forest. They noticed that when females have a young elephant, they shift their diet towards an increased consumption of supposedly medicinal plants, from which according to them the calf benefits when it consumes its mother’s dung. This remark is thus in line with the justifications for the therapeutic efficacy of EF given by the mahouts interviewed in Thongmyxay.

Thus, although a minority of mahouts (28% of those who reported therapeutic uses of elephant faeces) put forward explanations for the therapeutic efficacy of elephant faeces, the elements on which these justifications are based (elephants eat many different plants including medicinal plants -*'phuet pen ya-'* that are found in their faeces or in EBC) are nevertheless known and shared by most of them. These elements, associated with the fact that they observe elephant calves consuming their mother’s faeces, and that in Thongmyxay village elephants still feed into the forest where they eat wild plants invested in Laos with specific virtues, may all contribute to a positive perception of this matter and to an implicitly recognized value of their therapeutic use.^4^

We have seen in Sect. 1 that the therapeutic justifications put forward by the users or prescribers of animal materials reported in zootherapy studies fall in the domain of the symbolic. In Laos the elephant is invested with an eminent symbolic dimension which is certainly no different to the perception that mahouts have of this animal in Thongmyxay and to the value associated with its products. But the explicit justifications provided by mahouts regarding the efficacy of the therapeutic use of elephant faeces contrast sharply with this type of justification of a symbolic or analogous nature. Instead of being based on an identified “signature” or symbolic virtue attributed to the elephant which would act in sympathy on the cured ailments, they emphasise the virtues of the various plant substances with potentially curative properties contained in the elephant faeces (or in the elephant dung beetle brood chambers made from them).

### Therapeutic potential of urines

Cow excreta *(bos indicus)* represent a significant part of the materials used in Ayurvedic medicine and the properties of cow urine have been particularly studied in India. Cow urine is rich in fatty acids and phenolic acids and has a high antioxidant potential [78–80]. The anti-microbial activity of cow urine has been tested in vitro on strains of pathogenic bacteria [80–83]. In vivo, immuno-stimulant properties have been demonstrated in rats [84, 85]; one study showed that external application of cow urine hastened the healing process on wounded rats [86]. A study has shown anti-diabetic activity of cow urine in rats with diabetes induced by streptozotocin injections: it significantly increases their glycogen levels and reduces their blood glucose levels [87]. In humans, a clinical assay of a cow urine extract given orally has shown to significantly relieve patients suffering from haemorrhoids [88]. 

In the Middle East camel excreta are traditionally used to treat various ailments and the urine of this animal has also been the subject of numerous pharmacological studies [89].^5^ Anti-microbial properties of camel urine have been demonstrated in vitro showing antifungal activity on *Candida albicans* and *Aspergillus nige* and anti-bacterial activity against multi-drug resistant pathogenic bacteria [90, 91]. Alyahya et al. [92] showed in vitro anti-coagulant activity of camel urine, which significantly inhibits human platelet aggregation responses. In vitro studies have highlighted the potential of camel urine to inhibit the growth of tumours by limiting tumour angiogenesis and by their cytotoxicity against various human cancer cell lines. Anti-tumour and anti-metastatic activity of this urine has been shown in vivo in mice [93–96]. The composition of urine varies from one mammal species to another, but also according to the physiological state of the individual and their diet [97]. Thus, Wu et al. showed in vitro the anti-tumour activity conferred on mouse urine when collected after sustained physical effort [98].

Human urine, which is used in various traditional pharmacopoeias (see Sect. 2.2), has been the subject of several studies highlighting its therapeutic properties: in local application on wounds inflicted on previously anaesthetised animals, the healing properties of this urine have been shown in vivo on rats [99], and Khan & al. showed its emollient and healing effect on skin abrasions in rabbits [100]. Consumption of one’s own urine (auto- or self-urine therapy) is considered in Hindu tradition to be particularly beneficial to health and thus likely to increase the longevity of its followers [101, 102]. It has been popularised outside India through the publication of a book de dedicated to this practice and regularly re-edited since 1944 [103]. Auto-urine therapy was the subject of several clinical studies in the first half of the last century, dealing with endocrine problems and various inflammatory or auto-immune diseases [104]. Cases of regression of tumours following diversion of the patient’s urinary tract to the intestine have led to the hypothesis of an anti-tumour activity of the patient’s own urine [101]. Eldor suggested that the many tumour antigens present in the urine of a cancer patient are transmitted to the intestinal lymphatic system, which may produce specific antibodies that allow the body to destroy its own tumour cells [104]. Finally, human urine is currently being investigated as a potential non-invasive source of human stem cells for tissue culture and regenerative therapies [105].

In our study two mahouts used elephant urine and one mahout used human urine in veterinary preparations to treat skin problems in elephants (abrasions and rashes), another mahout reported the use of elephant urine in case of serous otitis and four others reported that people with diabetes have asked them for elephant urine to improve their condition. No studies have yet been carried out on the properties of elephant urine, but the use of this urine in Xayabury province is consistent with the above-mentioned studies highlighting the anti-infectious, emollient and healing potential of various mammalian urine, as well as the anti-diabetic potential of cow urine.

### Therapeutic potential of faeces

#### Substances present in the faeces

The therapeutic properties of the faeces of some animal species have been studied in Korea and China. The use of silkworm faeces (*Bombyx mori*) to strengthen internal organs, protect against diplegia, and treat diabetes [106] is recorded in the Dongui Bogam, a treatise on traditional Korean medicine dating from the fifteenth century. Hwang et al. [107] similarly mention that they have been used in China for palsy, blood circulation problems, fever, unhealthy eyes, headache, itching, and arthritis. The anti-allergic potential of these faeces and its *modus operandi* has been studied by Jung et al. [106]. Their fibrinolytic properties were highlighted and studied (Ahn et al. 2002) as well as their cytotoxicity on colon cancer cells [107, 108]. The ‘Wu-Ling-Zhi’, a preparation derived from faeces of the complex-toothed flying squirrel (*Trogopterus xanthipes*) is used in Chinese traditional medicine to treat stabbing pain in the chest and hypochondrium, as well as dysmenorrhea, amenorrhea, swelling and aching due to traumatic injury, postpartum blood stasis, and snake bites [109]. Extracts of this faeces have been shown to inhibit gastric acid secretion and to have a protective effect on gastric mucosa in vivo on rats [110]. Many compounds (terpenoids, flavonoids, lignans, sterols, esters and bile acids) known for their wide spectrum of pharmacological activities such as anti-inflammatory, anti-cerebral ischemia, anti-gastric ulcer, and anti-thrombin effects have been isolated from the faeces of this animal [109]. Calcined wild boar *(Su scrofa)* faeces is a preparation of traditional Tibetan medicine used to treat stomach and gallbladder diseases, such as dyspepsia, anorexia, jaundice, gallstones and nausea *(ibid)*. The same publication reports various Chinese pharmacological studies that have highlighted the presence of bile acids in this material, its high Ca, K, Mg, and Fe content, and have shown that this matter prevents mucosal damage caused by experimental colitis in rats, and significantly reduces the damage of colonic mucosa congestion, hyperplasia and ulcer.

In a zootherapy study carried out in Sudan, the author raises a hypothesis to account for the effectiveness of camel faeces (*Camelus dromedarius*) traditionally used to relieve arthritis, saying that “this property may be attributed to the active constituents present in herbs on which the camels feed”, but without giving further indications on the plants composing the diet of this animal in the area considered, and their potential or possible therapeutic uses [111]. This hypothesis is in line with the opinions expressed by the mahouts we have interviewed, who attribute the therapeutic value of EF and EBC to the fact that elephants consume a large number of species, including medicinal plants found in their dung. We provide the following facts in support of this assertion:

- With regard to the diversity of elephant diets, the inventories of plants consumed by *Elephas maximus* carried out in Asia [112–117] and the one we conducted with mahouts in Xayabury province, which includes 114 species collected, constituting 112 ethnospecies (cf. Additional file 2: Plants species consumed by elephants) attest to the wide range of species making up the diet of elephants in Asia.
- On the presence of medicinal plants in the elephants’ diet, we interviewed four traditional healers (one female and three males) in Thongmyxay district. None of them used animal materials for healing, nor were they aware of the local use of elephant excreta, but we presented them with the list of plants composing the diet of elephants which we inventoried with the mahouts and asked them which of these they used (see Additional file 2). Out of the 112 ethnospecies presented, 72 are used by these healers to treat various problems affecting humans, and two to heal buffaloes. These 74 species thus represent 66% of species of the elephant diet known to the mahouts of Thongmyxay. These results and the origin of their knowledge will be analysed in more detail in a dedicated study, but what we will note here is that, as pointed out by some mahouts, the elephant’s diet in Thongmyxay does indeed include a significant number of medicinal plants used locally. Similarly, a recent study carried out in northern Thailand analysing species overlap (based on data combining fieldwork survey and other published dataset) between Karen ethnoveterinary plant use for elephants, Karen medicinal plant use and Asian elephant diet, indicates that 84% of the species with ethnoveterinary uses among Karen are known to be consumed by *Elephas maximus* in Asia [118].

The low performance of the digestive process in elephants, which are post-gastric herbivores, leaves a substantial mass of non-processed plant residues in their faeces (according to Sukumar [119], only 40–50% of the forage consumed by *Elephas maximus* is actually digested), giving them there very fibrous texture.

It is thus likely that a significant proportion of secondary plant metabolites are found intact in their faeces. It is also possible that some metabolites of plant items consumed by elephants, which are neutral to human physiology or cannot be assimilated, become bioactive during the transformation that takes place through the elephants’ digestive process (the so-called prodrugs). It would therefore be interesting to carry out a metabolomic study on the faeces of elephants feeding in their natural environment, in order to highlight the possible presence of metabolites with recognized pharmacological activity in the samples collected, and to assess the therapeutic potential of these faeces from this point of view. Particular attention could be paid to metabolites likely to account for the therapeutic effects attributed to these faeces in the main indications for which they are used (Table 2). Such investigations could be performed on samples taken at different times of the year marking variations in the elephant's diet.

Regarding the use of EBC, although mahouts do not seem to attribute different properties to them than to EF (apart from the fact that this elephant material has been fragmented by the elephant dung beetle), it is possible that the faeces of the larvae that feed on the chamber and accumulate in it also contribute to the therapeutic potential of this material.

#### Potential of the microbiota present in the faeces

The therapeutic use of microbial material from healthy individuals is gaining new momentum with recent research on the human microbiota. It is practised in the form of faecal matter transplantation (FMT), which Brandt [120] defines as “the infusion of a faecal suspension from a healthy individual into the GI tract of another person to cure a specific disease”. Zhang links this practice to a similar form of therapy attested as early as the turn of the fourth century AD in the Zhou Hou Bei Ji Fang, a treatise on traditional Chinese medicine that mentions the use of a suspension of human faeces by oral intake [7]. It appears to have been subsequently used in China to treat disorders defined in the emic terms of the Chinese medical tradition, disorders that cover febrile disease, typhoid fever and diarrhoea, poisoning, abscess, food stagnation, and was still recently used in China by some elder-generation physicians for refractory diseases [121]. A practice similar to FMT is also found in Europe in the veterinary care of ruminants, in the form of a transfer of the contents of the digestive tract of a healthy individual taken during its bolus regurgitation to an animal that has lost its digestive capacity. This practice was reported in the seventeenth century by the Italian anatomist Fabricius Aquapendente: “I have heard of animals which lose the capacity to ruminate, which, when one puts into their mouths a portion of the materials from the mouth of another ruminant which that animal has already chewed, they immediately start chewing and recover their former health” (Quoted from Borody [122], his translation). This practice is similarly attested in the eighteenth century in Sweden [123]. A similar technique, used under the name ‘transfaunation’ was employed to study the hindgut fauna of termites and was published in 1927 [124]. The term transfaunation has since been retained to refer to today’s common veterinary practice of treating ruminants with severe disorders due to disturbance of the rumen microbiota, by inoculating them with rumen material from healthy congeners [123] but also in the word compound 'fecal transfaunation' as a synomnym of FMT. FMT is also practised on horses via the naso-gastric or rectal route for the treatment of certain infectious diarrhoea [125, 126], and has been reported to have been used on an Asian elephant female following an intestinal obstruction [127]. In modern human medicine, FMT was revived by Eiseman in the 1950s [128] to treat pseudomembranous colitis due to infestations of the colon with pathogenic bacteria favoured by antibiotic therapy. It was subsequently extended to the digestive decolonisation of multi-resistant bacteria [129], particularly in immune-compromised subjects at greater risk (HIV infection, chemotherapy, immunosuppressive treatment) to restore a disturbed microbiota [130, 131]. With the recent development of knowledge on the involvement of the intestinal microbiota in many physiological processes, research on the applications of FMT has extended to multiple indications. Among these are functional disorders and chronic inflammatory bowel diseases [132, 133], certain forms of autism, epilepsy and Parkinson’s disease—which could be linked to the diffusion of neuro-metabolites produced by the digestive microbiota [134, 135], obesity, chronic fatigue syndrome, multiple sclerosis, myoclonus dystonia, insulin resistance and the metabolic syndrome [120]. This brief overview of the wide range of applications of FMT thus echoes the multiple indications of animal or human faeces administered orally, found in the URs in Additional file 1:URs of animals excreta (34 records).

The uses of elephant faeces recorded with the mahouts fall mainly into two groups of indications: gastro-intestinal disorders on the one hand and dermatological problems on the other. With regard to gastro-intestinal disorders, we can consider in the light of the studies we have just mentioned, the hypothesis of a curative action of these faeces by rebalancing the user’s microbiota via a supply of bacterial material from the elephant’s microbiota. In these uses, the oral route used by mahouts is comparable to that reported by Fabricius Aquapendente in ruminants, or to the naso-gastric route used in hospitals on patients suffering from severe diarrhoea who would not hold a suspension administered by enema, or on patients who have already been intubated [136, 137].^6^ While an aqueous maceration of EF or EBC is used in three of the URs reported by mahouts or their families, in nine others a decoction is used, raising in the latter case the question of whether the ingested bacterial material can retain a curative potential. However, as the faeces or EBC used are usually dried beforehand, these materials may contain sporulated bacteria that are resistant to this method of preparation, as is the case for other dried animal matter [138]. For indications concerning skin problems such as fungus and certain inflammations, these conditions can also be caused by a disturbance of the skin microbiota. But in these applications the hypothesis of a possible rebalancing of a disturbed microbiota by an external supply of microbial material is less plausible because of the aerobic nature of the cutaneous microbiota whereas the material applied here comes from the internal anaerobic microbiota of the elephant. For EF’s internal use in the case of these dermatological indications, on the other hand, this hypothesis could account for a curative effect on metabolic disorders that result in skin rashes.

### Zoonotic risks

The use of animal materials, and in particular body fluids, raises the question of the risks of zoonotic transmission of pathogens, a risk that has become particularly acute in the current context of increasing zoonotic pandemics.

*(a) Urine:* This fluid resulting from the filtration of blood by the kidneys is normally free of pathogens, but cases of contamination of goat and cow urine have nevertheless been reported [139] and urine can also carry germs in the case of urinary tract infections. The therapeutic use of urine therefore requires caution and discernment. A case of vision loss is reported as a result of a man’s self-application of his own urine to treat an inflammation of the eyes, but it is not specified whether this loss is due to an infection following this treatment or to the action of deleterious compounds in the urine used. [140].

*(b) Faeces:* The microbial flora of animal faeces is far from being free of potential human pathogens [141–143], and thus their handling and application especially on wounds (6 UR in Additional file 1: URs of animals excreta) or as a haemostatic in case of bleedings (8 UR) presents a non-negligible risk of infection; in addition some topical applications may also be associated with incisions [18]. In one UR (cf. Additional file 1) faeces of the palm civet *(Nandinia binotata)* mixed with water are used as an enema for a baby suffering from stomach pain [18]. Regarding intakes through oral route, 36 UR of faeces uses in the literature consulted (cf. Additional file 1) and 15 UR we recorded from mahouts can be considered to fall within the therapeutic field of FMT, since they consist (except in one case) of an oral transfer of faecal material from one individual to another, but with the clear difference that these transfers are in this case interspecific (35 animal-to-human UR and one donkey-to-dromedary UR listed in Additional file 1). These therapeutic uses of animal faeces potentially involve risks of zoonotic transmission of pathogens. In Thongmyxay district, where we found a relatively large and homogenous corpus of EF uses, it would therefore be appropriate to carry out research comparing the intestinal microbiota of wild and village elephants, of mahouts who are in contact with the latter, and of people from the same region who have or have not used this type of treatment. This would allow to highlight possible exchanges of microbial and parasitic material between men and village elephants and to estimate the potential risks of pathogen transmission between elephants and humans,^7^ and also between village elephants and their wild counterparts: village elephants from Thongmyxay are indeed periodically released in the surrounding forested area which shelters the second largest population of wild elephants in Laos, and village elephant cows sometimes mate with wild bulls [27], whereas transmissions through faecal material are potentially favoured by the allocoprophagous behaviours of the calves. Such a study could also provide elements to evaluate the therapeutic potential of EF medicinal uses from the point of view of the transfer to humans of the residual microbiotic material still contained in the EF used.

The Covid 19 pandemic we are currently experiencing may be of zoonotic origin, perhaps involving contact with animal faeces. The closest genetic relative of the coronavirus involved was found in bat species [147]. The flesh or faeces of diverse bat species are used in traditional therapeutic applications in Laos, China and north-eastern India [31, 52, 109, 148], and bat guano is also used as a fertilizer in Southeast Asia [149, 150]. Even in the case of an emerging pathology, traditional medicines do not seem any more deprived than modern biomedicine to react to a new problem, such as seen with the examples given by William [151] where the scope of existing resources is extended to new indications. Thus the Reuters news agency and the Namibian press reported in August 2020 an extension of the indications for the use of elephant faeces, traditionally used according to these sources for nose bleeds, headaches, toothaches, and other types of pain, to treat people affected by the COVID 19 pandemic [77, 152]. Despite a public denial of this rumour regarding the effectiveness of elephant faeces against this infection by the country’s Minister of Health (without any argumentation, however, reported by sources citing this denial), the market for this material was then booming [77]. A medicinal products salesman interviewed by a journalist reported that elephant dung is then used in saunas, claiming a decongestant effect which helps with breathing, coughing and runny nose, and can provide relief in case of colds and flus (*ibid*). If this hypothesis is after all consistent with the mode of administration evoked, however, this practice is not exempt of potential risks of contamination as shown by a severe case of pulmonary anthrax reported in Pennsylvania, following the inhalation of *Bacillus anthracis* spores during contact with dried animal skins [138].

## Conclusion

The uses of elephant faeces and urine that we have documented in the province of Sayaboury in Laos are part of a widely spread tradition over time and space of therapeutic uses of animal or human excreta, both in the scholarly medical traditions and in oral medical traditions from very diverse cultures and geographical origins. These still widespread uses, which involve the handling of animal faeces, their ingestion or application to different parts of the body, and sometimes lead to their sale in medicinal markets, represent potential sources of pathogen transmission from animals to humans. In the context of the globalisation of trade that currently prevails and which favours the emergence of zoonoses, it becomes crucial to further document the zootherapy practices in use in the world and to know the species mobilized in these practices. This will allow to assess the potential zoonotic risks inherent to these uses, and help to quickly grasp the possible sources of contamination that may be at the origin of an epidemic when it breaks out.

In the One Health concept perspective, the therapeutic uses of faeces reported worldwide, as well as the allocoprophagic behaviours of juvenile domestic animals, must be considered from the point of view of a compromise to be found between the infectious risk inherent in these uses or behaviours and the potential benefits that they may provide for heath or, in the field of animal husbandry, for the development of healthy individuals [75].

The therapeutic indications for which elephant urine is used in Xayaboury province are consistent with the properties and curative potential of the urine of other species highlighted in the biological and biochemical studies performed on these fluids.

With regard to the uses of EF that we have recorded, the justifications provided by mahouts for their therapeutic efficacy contrast with the justifications of zootherapeutic practices generally reported in studies devoted to this field, since they are not based on a “signature” recognised in the elephant which would act in sympathy with the treated ailments, but on the intrinsic virtues of the various plant substances contained in its faeces. These justifications are supported by the great diversity of species making up the elephants’ diet, which argues for the interest of carrying out a metabolomic analysis of faeces of elephants feeding in their natural environment in order to assess their therapeutic potential.

An interesting feature in these zootherapeutic uses of EF highlighted by the therapeutic justification of mahouts for their use, is that here it is not so much the raw material that is considered but rather the animal contribution to its processing through the elephant ability acknowledged by mahouts to select valued plants from the wild, making its faeces a kind of "medicinal cocktail".

## Supplementary Information

**Additional file 1.** URs of animal excreta in zootherapic studies.

**Additional file 2.** Inventory of plants consumed by elephants.

## Data Availability

All data generated or analysed during this study are included in this published article and its supplementary information files].

## Abbreviations

EBC: Elephant dung beetle brood chamber
ECC: Elephant conservation center
EF: Elephant faeces
NNPA: Namphuy national protected area
UR: Use report

## Footnotes

1 Also noteworthy is a review on the therapeutic uses of human urine in Spain that lists more than 200 URs throughout the country [15], which, with the exception of the references quoted in this review that can be consulted online, we have not retained due to the lack of access to most of the sources mentioned, many of which written in Spanish come from publications of limited diffusion, and whose remarkable exhaustivity would have over-represented the use of human urine in this brief overview intended to give an idea of the extent of these zootherapeutic practices.

2 Meyer-Rochow (2017), taking the etic point of view of the biomedicine, insists that although some uses may seem ridiculous and superstitious, their real therapeutic potential should not be dismissed and should be examined from the point of view of the properties of the substances handled and used. [19].

3 This considerable quantity of buried elephant faeces, added to that consumed by adult individuals, shows the significant ecological role played by these insects in accelerating the decomposition of the excrement produced by elephants, as well as by the digging of tunnels and brood chambers which contribute to the aeration of the soil and the percolation of rainwater into the ground.

4 It is worth noting that the same argument for the effectiveness of this material is reported by a journalist regarding the therapeutic use of elephant faeces in Namibia: " Elephants are herbivores, and their dung is said to have medicinal properties because of the combination of herbs, seeds and plant matter they consume."[77].

5 These studies do not specify which camelid species is involved (*Camelus dromaderius* or *bactrianus*), but according to the information given by Alkhamese it is most probably *Camelus dromedarius*, widely domesticated in North Africa and the Middle East.

6 In Brumbaugh’s study faecal material is also administered orally in the form of capsules, but it is not specified whether their shell is gastro-resistant.

7 In human to elephant transmission, the possibility of 'reverse zoonosis' has been discussed in relation to the presence of the human tuberculosis germ *Mycobacterium tuberculosis* detected in wild and captive elephants in Asia and in captive elephants in the USA and France [144–146]; for elephant to human, in the context of the therapeutic use of EF in Xayabury province, elephant dung of a rather solid and fibrous texture is collected and handled fresh even if theoretically used dried.
