# Supplementary material for: Zootherapeutic uses of animals excreta: the case of elephant dung and urine use in Sayaboury province, Laos
Source: J Ethnobiol Ethnomed. 2021 Oct 28;17:62. doi: 10.1186/s13002-021-00484-7 (PMC8552211; doi:10.1186/s13002-021-00484-7)
Supplement: Supplementary file 1 — Additional file 1. URs of animal excreta in zootherapic studies. [file 13002_2021_484_MOESM1_ESM.docx]

**Appendix 1**

Contemporary use reports of animal faeces or urine recorded in zootherapy studies

| **Pays** | **Animal** | **Part used** | **Indication and administration** | **Administration** | **Ref.** |
| --- | --- | --- | --- | --- | --- |
| Albania | Dog (Canis lupus familiaris) | Faeces | Hepatitis: faeces mixed with flower and baked into a small bread to consume | oral | [50] |
| Albania | Hen (Gallus domesticus) | Faeces | Amulet against the Evil made with dried faeces is hung on dress. | worn | [50] |
| Albania | Human (Homo sapiens) | Faeces | Eye inflammation: fresh child feces are externally applied | topical | [50] |
| Albania | Human (Homo sapiens) | Faeces | Snake bite: fresh feces are applied to the bite | topical | [51] |
| Albania | Donkey (Equus asinus) | Urine | Sinusitis: instilled in the nose (urine has to come from young animals only) | topical | [49] |
| Albania | Human (Homo sapiens) | Urine | Hepatitis: drunk fresh | oral | [50] |
| Angola | African elephant (Loxodonta africana) | Faeces | Rheumatism: smear on affected part Difficult births, Treat sick animals:  decoction to drink | oral, topical | [13] |
| Angola | Spotted hyena (Crocuta crocuta) | Faeces | Epilepsy, malaria: faeces mixed with water to drink | oral | [13] |
| Brazil | Dog (Canis lupus familiaris) | Faeces | Measles, menstrual cramps | ns | [4] |
| Brazil | Parrot (Amazona sp.) | Faeces | Earache, alcoholism | ns | [4] |
| Brazil | Boa constrictor | Faeces | ns | ns | [5] |
| Brazil | Dog (Canis lupus familiaris) | Faeces | measles, tea | oral | [6] |
| Brazil | Hen (Gallus domesticus) | Faeces | Ethnoveterinary use for mastitis, furunculosis | ns | [9] |
| Brazil | Male Goat (Capra hircus) | Faeces | Ethnoveterinary use for Feces pointed stakes | ns | [9] |
| Brazil | Dog (Canis lupus familiaris) | Faeces | Sun-dried faeces are recommended against chicken pox | ns | [17] |
| Brazil | Gray rocket deer (Mazama gouazoubira) | Faeces | Headache | ns | [18] |
| Brazil | Human (Homo sapiens) | Urine | Ethnoveterinary use for Intoxication of the cattle when the ones feed of cassava: a cup of human urine in 3 liters of water, is given to the cattle to drink after the plant ingestion | oral | [9] |
| Brazil | Striped hog-nosed skunk (Conepatus semistriatus) | Urine | Backpain | ns | [18] |
| Cambogia | Water buffalo (Bubalus bubalis) | Faeces | Burn: faeces applied directly on the burn or mixed with Luffa aegyptiaca (leaves) and applied | topical | [15] |
| Cambogia | Human (Homo sapiens) | Urine | Use human urine on a wound  or crush the leaves of Chromolaena odorata and mix with urine for applying on the wound. | topical | [14] |
| Canary Islands | Dog (Canis lupus familiaris) | Faeces | Spider bites: powdered faeces are ingested | oral | [57] |
| Ethiopia | Ethiopian hare (Lepus fagani ) | Faeces | Soars/wound: Excreta are used as ointment | topical | [36] |
| Ethiopia | Human (Homo sapiens) | Faeces | Wart: used as oinment | topical | [36] |
| Ethiopia | African elephant (Loxodonta africana) | Faeces | Migraine headache: faeces are used with water | ns | [63] |
| Ethiopia | Goat (Capra hircus) | Faeces | Dandruff: fresh or dry fecal matter is used with water | topical | [63] |
| Ethiopia | Chicken *(Gallus gallus)* | Faeces | Combining excrement with mud and applied to infected skin | topical | [68] |
| Ethiopia | Goat *(Capra hircus)* | Faeces | To fight dandruff: dried, powdered, mixed with water, and smeared over the head | topical | [68] |
| Ethiopia | Lizars (Lacerta spp) | Faeces | Skin problems: pounded fecal matter, dried and with secret ingredients applied to swellings | topical | [68] |
| Ethiopia | Monkey *(Papio anubis)* | Faeces | Sleeping sickness: Dried fecal matter is fumigated | fumigation | [68] |
| Ethiopia | Cow (Bos taurus) | Urine | Malaria : urine is drank | oral | [36] |
| Ethiopia | Gazelle (Gazella spp.) | Urine | For urination problem: urine is drank | oral | [36] |
| Ethiopia | Cat (Felis domesticus) | Urine | Goiter: fresh urine used | ns | [63] |
| Ethiopia | Cow (Bos taurus) | Urine | Malaria: fresh urine is used | ns | [63] |
| Ethiopia | Red deer (Cervus elaphus) | Urine | Urinary retention: fresh urine is used | ns | [63] |
| India | Dhole (Canis alpinus) | Faeces | stomach poisoning - oral | oral | [7] |
| India | Goat (Capra indica) | Faeces | gout- topic onguent | topical | [7] |
| India | Hare (Lepus nigrocolis) | Faeces | Fit (cough): smoke inhalation | inhalation | [7] |
| India | Indian bison (Bos gaurus) | Faeces | Promote hair growth - Topic (Dry dung boiled with coconut oil) | topical | [7] |
| India | Rat (Rattus rattus) | Faeces | Urine obstruction - Topic (abdomen) | topical | [7] |
| India | Cow (Bos indicus) | Faeces | Skin disease cowdung is mixed with crushed powder formed of the root of Borborial tree (Sida rhombifolia) and applied externally | topical | [12] |
| India | Asian elephant (Elephas maximus) | Faeces | Skin infection, easy teething in kids - teeth paste with honey and borax on stone- applied over gums | oral | [21] |
| India | Camel (Camelus dromedarius) | Faeces | Ash of burnt dung are applied on stomach to treat constipation | topical23] | [28] |
| India | Dog (Canis lupus familiaris) | Faeces | Wounds: excreta is applied | topical | [28] |
| India | House sparrow (Passer domesticus) | Faeces | Ash of excreta are used for treatment of asthma in children | ns | [28] |
| India | Zebu (Bos indicus) | Faeces | Ash of burnt dung are applied on stomach to treat urticaria | topical | [28] |
| India | Human (Homo sapiens) | Faeces | Faecal material is taken orally to induce vomiting during stomach poisoning. | oral | [29] |
| India | Camel (Camelus dromedarius) | Faeces | constipation: dung is used for constipation | ns | [30] |
| India | Crow (Corvus splendens) | Faeces | Blister (ulcer on body): Excreta are topically applied | topical | [30] |
| India | Cow (Bos taurus) | Faeces | Ethnoveterinary use for elephant swelling : Rice flour is mixed with salt and cow dung and is applied | topical | [31] |
| India | Goat (Capra hircus) | Faeces | Cuts, burns : Fresh excreta along with water are used on cuts and burns Early detachment of infant umbilical cord or if detached cord is infected: fire-dried excreta are crushed, powdered and applied on the navel of the newborn infant | topical | [32] |
| India | Goat (Capra hircus) | Faeces | Inflammations | ns | [35] |
| India | Goat (Capra hircus) | Faeces | Inflammations | ns | [35] |
| India | Cow (Bos indicus) | Faeces | Muscle pain can relieve by smear of dung and milk mixture | ns | [40] |
| India | House sparrow (Passer domesticus) | Faeces | Fecal matter is applied in the anus of baby to treat constipation | topical | [40] |
| India | Indian ass (Equs hemionus) | Faeces | Dung kept in water and after one day filtered water is given to cure jaundice. | oral | [40] |
| India | Camel (Camelus dromedarius) | Faeces | Stomachache: taken with water | oral | [43] |
| India | Pigeon (Columbo libea) | Faeces | Typhoid : Mixed with rice beer and consumed | oral | [42] |
| India | Porcupine (Hystrix indica) | Faeces | Stomach disorders (children), asthma: stomach and intestinal parts are dried (along with the fecal matter) soaked overnight and taken orally | oral | [45,46] |
| India | Cow (Bos taurus) | Faeces | Hair growth: dry dropping boiled with coconut oil and massge the hair | topical | [46] |
| India | Goat (Capra indica) | Faeces | Arthritis: smooth paste with water is applied on swelling | topical | [46] |
| India | Hare (Lepus nigrocolis) | Faeces | Fitness: fume of droppings is taken nasaly | inhalation | [46] |
| India | House sparrow (Passer domesticus) | Faeces | Constipation: applied in the anus of baby | topical | [46] |
| India | Zebu (Bos indicus) | Faeces | Muscle pain: can be relieved by smear of milk and dropping mixture | topical | [46] |
| India | House sparrow (Passer domesticus) | Faeces | Constipation: bandage | topical | [52] |
| India | Cow (Bos Taurus) | Faeces | Epistaxis: gases of fresh dung is inhale once daily for 6 days | topical | [54] |
| India | Pigeon (Columbo libea) | Faeces | Typhoid, toothache: Mixed with rice beer and consumed | oral | [59] |
| India | Buffalo (Bos bubalus) | Faeces | Eczema: curd is mixed in the dung and applied locally | topical | [59] |
| India | Zebu (Bos indicus) | Faeces | Sprains: heated and applied topically Cough, baldness: rubbed on externally | topical | [60] |
| India | Donkey (Equus asinus) | Faeces | Ethnoveterinary use for camel diarrhoea: grinded and dissolved in water given to drink | oral | [61] |
| India | Crow (Corvus splendens) | Faeces | Feces or excreta of dead crow: tumor on body feces or excreta rubbed on tumor | topical | [62] |
| India | Common pigeon (Columba livia) | Faeces | Dracunculiasis (guinea-worm disaese): Excreta or faeces mixed with honey and used as tablets | oral | [62] |
| India | Dog *(Canis familiaris)* | Faeces | Droppings of dog are taken orally to induce vomiting during stomach poisoning | oral | [64] |
| India | Hare Lepus nigricollis | Faeces | Direct excreta mixed with coconut oil are used to treat ringworm | ns | [64] |
| India | Indian bison *(Bos gaurus)* | Faeces | Dung boiled in coconut oil promotes hair growth | ns | [64] |
| India | Goat (Capra indica) | Urine | tuberculose- oral | oral | [7] |
| India | Ass (Equus asinus) | Urine | eye care: used as eye liner for eye power | topical | [7] |
| India | Cow (Bos spp.) | Urine | urine is used as an eye drop | topical | [7] |
| India | Goat (Capra indica) | Urine | Tuberculosis: urine is administered orally,2 teaspoons 21 days regularly | topical | [7] |
| India | Mainland Serow (Capricornis sumatraensis) | Urine | Diabetes: 5 ml of fresh urine is taken once a month | oral | [11] |
| India | Indian rhinoceros (Rhinoceros unicornis) | Urine | Jaundice: urine is prescribed to drink. | oral | 12] |
| India | Barking deer (Muntiakus munjak) | Urine | Otorrhoea: urine is dropped into the ear | topical | [16] |
| India | Indian bison (Bos gaurus) | Urine | Scurvy at thighs and legs: first urine is collected and taken orally | oral | [16] |
| India | Goat (Capra sps.) | Urine | Urine is taken to cure paralysis, tubercolosis, asthma, skin diseases and stomachache | ns | [19] |
| India | Human (Homo sapiens) | Urine | Applied to eye injuries, as antiseptic on flesh wounds. | topical | [19] |
| India | Cow (bos indicus) | Urine | Skin problem on hands, Applied over the affected area | topical | [26] |
| India | Dog (Canis lupus familiaris) | Urine | Urine cures earache and is applied on fore head to relieve headache. | topical | [29] |
| India | Goat (Capra sibirica) | Urine | Urine is taken to cure paralysis, tuberculosis, asthma, skin diseases and stomachache. Also cures night blindness, jaundice and other liver related problems. | oral | [29] |
| India | Human (Homo sapiens) | Urine | Urine is applied on eye injuries. Also applied as antiseptic on fresh wounds. Faecal material is taken orally to induce vomiting during stomach poisoning. | topical | [29] |
| India | Cow (Bos taurus) | Urine | Wound healing: Urine or saliva is directly applied on cut area | topical | [30] |
| India | Human (Homo sapiens) | Urine | Wound healing/ injury due to cut: directly applied it is an antiseptic. | topical | [30] |
| India | Flying squirrel (Biswamoyopterus biswasi) | Urine | The urine is believed to disolve stone particles inside gall bladder and kidney of human sufferer. Rice grains are stored inside the bladder which is smoked and dried. 2–3 rice grains after drying are consumed along with water whenever required. | ns | [32] |
| India | Human (Homo sapiens) | Urine | Toothache: applied on teeth to cure toothache; Burns: mud from urinated on mud is applied on cuts and burns Cunjunctivitis: urine is applied to eyes | topical, oral | [32] |
| India | Flying squirrel (Pteromys sp.) | Urine | Urethritis: Urine is orally administered (or) mix with rice for preservation powdered and dissolved in water when taken. | oral | [33] |
| India | Musk deer (Maschus sp.) | Urine | Ear swelling and pain: urine is used as ear drops | topical | [33] |
| India | Flying fox (Eropus sp) | Urine | Kidney stones: soaked in rice, dried and taken | oral | [34] |
| India | Goat (Capra sibirica) | Urine | Asthma tuberculosis, paralysis, skin disease, stomach disorder | ns | [34] |
| India | Goat (Capra hircus) | Urine | Tuberculosis | ns | [35] |
| India | Human (Homo sapiens) | Urine | Wound healing | ns | [35] |
| India | Barking deer (Muntiacus munjak) | Urine | Ottorrhaea: urine inside gallbladder dropped into the ear. | topical | [38] |
| India | Cow (Bos indicus) | Urine | Weakness due to fever is cure by drinking urine, given to cure cancer | oral | [40] |
| India | Dog (Canis familiaris) | Urine | Used as eardrop for curing earache | topical | [40] |
| India | Human (Homo sapiens) | Urine | Human urine is used as antiseptic for wound healing | topical | [40] |
| India | Goat (Capra indicus) | Urine | Cough,,tuberculosis: urine of goat is administered orally | oral | [40][41] |
| India | Bail (Bos taurus indicus) | Urine | Earache: two to three drops of urine of cat is dropped in the ear in | topical | [44] |
| India | Billy (Prionalilurus viverrinus) | Urine | Earache: two to three drops of urine of cat is dropped in the ear in | topical | [44] |
| India | Gai (Bos indicus) | Urine | typhoid and jaundice: two or three sips of urine is given orally and remaining urine is applied on the whole body | oral | [44] |
| India | Goat (Capra indica) | Urine | Tuberculosis: taken orally | oral | [46] |
| India | zebu (Bos indicus) | Urine | Eye disease: urine is used as an eye drop | topical | [46] |
| India | Zebu (Bos indicus) | Urine | Fever, weakness: taken orally | oral | [46] |
| India | Cow (Bos Taurus) | Urine | Malaria:urine drunk | oral | [52] |
| India | Human (Homo sapiens) | Urine | Urine drop is applied twice daily for pile | topical | [54] |
| India | Goat (Capra capra) | Urine | Insect bite: urine of female goat mixed with 'kodapal patta' is consumed | topical | [56] |
| India | Cow (Bos indicu) | Urine | Anemia: fresh urine is taken | oral | [59] |
| India | Human (Homo sapiens) | Urine | Conjunctivitis,skin disease: fresh urine is taken | ns | [59] |
| India | Donkey (Equus asinus) | Urine | Amoebiasis : Fresh urine is boiled and consumed | oral | [59] |
| India | Human (Homo sapiens) | Urine | Rashes: applied on affected area | topical | [60] |
| India | Camel | Urine | Ethnoveterinary use for camel mastitis: collected and plastered with the soil for topical use | topical | [61] |
| India | Cow (Bos taurus) | Urine | Cough in children: Urine with sugar, equally mixed and drunk To purify blood and stomach: Urine, filtrate urine with cloth drunk | oral | [62] |
| India | Goat (Capra aegagrus ircus) | Urine | Urine cough, asthma: orally taken at morning | oral | [62] |
| India | Sheep (Ovis aries) | Urine | Cough (cold): urine drunk | oral | [62] |
| India | Goat *(Capra hircus )* | Urine | Tribals use goat urine orally in tuberculosis | oral | [64] |
| India | Human *(Homo sapiens)* | Urine | Milk and is used as eye drop in eye diseases | topical | [64] |
| India | Indian bison *(Bos gaurus)* | Urine | Urine is taken to cure paralysis. | ns | [64] |
| Italy | Cow (Bos taurus) | Faeces | Against whooping cough: smelling cow feces in the morning | inhalation | [48] |
| Italy | Dog (Canis lupus familiaris) | Faeces | Vulnerary for burns: dog feces are applied | topical | [48] |
| Italy | Human (Homo sapiens) | Urine | Anti-conjunctivitic: The first urine of the day is used to wash the affected eyes | topical | [51] |
| Italy | Human (Homo sapiens) | Urine | Haemostatic, disinfectant: human urine is used to rinse fresh bleeding lacerations | topical | [51] |
| Italy, Albania | Cow (Bos taurus) | Faeces | Vulnerary for burns: fresh cow feces are applied to the wound | topical | [48] |
| Laos | Bat (sp. not specified) | Faeces | part of mix to cure ear infections, part of mix to treat teeth ailments | ns | [8] |
| Laos | Asian elephant (Elephas maximus) | Faeces | Ethnoveterinary use for elephants wounds: A mixture of clay and elephant faeces is placed in a squame of banana stem and applied as a poultice | topical | [22] |
| Laos | Asian elephant (Elephas maximus) | Urine | diabetics boil 5 liter pots of elephant urine and ingest the medicine over a number of days to treat their conditions | oral | [8] |
| Laos | Human (Homo sapiens) | Urine | pain relief for people with serious internal injuries | ns | [8] |
| Laos | Asian elephant (Elephas maximus) | Urine | Ethnoveterinary use for elephants skin outbreak: chopped Alpinia galanga (L.) Willd. stems are mixed with elephant urine and applied on the affected area 2–3 times a day until healed | topical | [22] |
| Laos | Human (Homo sapiens) | Urine | Ethnoveterinary use for elephants skin outbreak: crushed fruit of Spondias pinnata combined with human urine is then applied to the skin | topical | [22] |
| Nepal | House & tree sparrow (Passer spp.) | Faeces | Feces of the bird are topically applied to the wound to accelerate the process of pus formation for quick healing. | topical | [39] |
| Nepal | Donkey (Equus asinus) | Urine | Ethnoveterinary use for sick livestock: Urine is given to ailing livestock to drink | oral | [51] |
| Nepal | Ovis ammon hodgsoni (Tibetan Argali) | Urine | Anti-otitic: Drops of urine are instilled in the affected ear | topical | [51] |
| Nigeria | African elephant (Loxodonta africana) | Faeces | Cure for cancer, treatment for pile, haemorrhage, headache | ns | [2] |
| Nigeria | Crocodile (Crocodylus niloticus) | Faeces | For spiritual protection | worn | [2] |
| Nigeria | African elephant (Loxodonta cyclotis) | Faeces | Fever mixed with local gin (distilled palm wine) and drank | oral | [25] |
| Nigeria | African elephant (Loxodonta cyclotis) | Faeces | Dysentery: mixed with tree bark and local gin (distilled palm wine) and drank | oral | [25] |
| Nigeria | African elephant (Loxodonta cyclotis) | Faeces | Epilepsy: mixed with herbs and rubbed all over the body | topical | [25] |
| Nigeria | African elephant (Loxodonta cyclotis) | Faeces | Swelling or bone problem: rub on swelling , rub on bone problem, or unknown ailments | topical | [25] |
| Nigeria | African elephant (Loxodonta cyclotis) | Faeces | Wound: add to snuff (tobacco) to seal wound | topical | [25] |
| Nigeria | African elephant (Loxodonta cyclotis) | Faeces | Mixed with water and rubbed on wound | topical | [25] |
| Nigeria | Buffalo (Syncerus caffer) | Faeces | Charm mixed with goat skin, herbs, and tied around the waist of a warrior as a bullet proof charm | worn | [25] |
| Nigeria | Dwarf Crocodile (Osteolaemus tetraspis) | Faeces | Swelling: ground and rubbed on affected area | topical | [25] |
| Nigeria | Leopard (Panthera pardus) | Faeces | Joint pain: ground with other herbs, mixed with palm kernel oil, and rubbed on affected part | topical | [25] |
| Nigeria | Palm civet (Nandinia binotata) | Faeces | Baby's stomach pain mixed with water and used as enema for baby | anal | [25] |
| Nigeria | Tortoise (Kinixya erosa) | Faeces | Rheumatism: collected, dried, mixed with alligator pepper until smooth, and applied on the portion affected after it has been pierced and little razor cuts have been made | topical | [25] |
| Nigeria | Monkey (Cercopithecus spp.) | Faeces | Cough: drank with water/ local liquor | oral | [25] |
| Nigeria | Cow (Bos taurus) | Faeces | Diabetes, haemorrhoid | ns | [47] |
| Nigeria | Aadvark (Orycteropus afer) | Urine | Cure of ear problem | ns | [2] |
| Nigeria | Cow (Bos taurus) | Urine | Convulsions, rheumatism, viral infection, general body pain | ns | [47] |
| Pakistan | Zebu (Bos indicus) | Faeces | Ethnoveterinary: Fresh dung boiled in Neem Oil with hinghot (Balanites aegyptiaca Linn.) is given to camels as an antihelmintic | ns | [37] |
| Pakistan | Zebu (Bos primigenius indicus) | Faeces | Ethnoveterinary: dung mixed in flowers of Rose (Rosa damascene Mill.) and Neem (Azadirachta indica) oil is used as a lotion in cases of dermatitis and as an acaricide in the treatment of mange. | topical | [37] |
| Pakistan | Nightingale (Luscinia megarhynchos) | Faeces | Feces of nightingale are useful in removing black spots of skin. | ns | [55] |
| Pakistan | Human (Homo sapiens) | Urine | Urine of male is splashed directly in the eyes of animals in case of conjunctivitis | topical | [37] |
| Pakistan | Human (Homo sapiens) | Urine | Urine is used as a haemostatic agent in case of wounds. | topical | [37] |
| Pakistan | Zebu (Bos indicus) | Urine | Ethnoveterinary: in case of foot rot in goats, hooves are dipped in the urine | topical | [37] |
| Phillipines | Goat | Faeces | Asthma; Stomach pain; Loose Bowel Movement: Seven pellets from the goat’s feces must be collected, toasted, pulverized, mixed with a glass of warm water and then drank | oral | [24] |
| Portugal | Common pigeon (Columba livia) | Faeces | Anti-asthma: the patient should eat pigeon feces | oral | [14] |
| Portugal | Cow (Bos taurus) | Faeces | Stops nosebleeds: cow excrement is inserted in the bleeding nose Against toothache: cow excrement is placed on the afflicted tooth | topical | [14] |
| Portugal | Dog (Canis lupus familiaris) | Faeces | Haemostatic: Toasted and powdered dog feces are applied to the wound | topical | [14] |
| Portugal | Donkey (Equus asinus) | Faeces | bleeding nose: donkey feces are smelled to stop the blood | topical | [14] |
| Portugal | Goat (Capra aegagrus hircus) | Faeces | Against roundworm: a mixture of goat’s feces is placed in the afflicted person’s eyes | topical | [14] |
| Portugal | Hen (Gallus domesticus) | Faeces | Against tumors: chicken excrement is placed over the tumor | topical | [14] |
| Portugal | House mouse (Mus musculus) | Faeces | Anti-colitis: a tea is made from mouse feces | oral | [14] |
| Portugal | Human (Homo sapiens) | Faeces | Against jaundice: human excrement covered in honey is applied over the afflicted area | topical | [14] |
| Portugal | Human (Homo sapiens) | Faeces | Heals tumors: human excrement is applied to the afflicted area | topical | [14] |
| Portugal | Sheeep (Ovis aries) | Faeces | bleeding nose: Sheep excrement is inserted into the nose | topical | [14] |
| Portugal | Timon lepidus (Ocellated lizard) | Faeces | Heals sore eyes, anticonjunctivitic: Lizard feces are applied to the eyes of humans and animals | topical | [14] |
| Portugal | Cow (Bos taurus) | Urine | Against cramping pains: cow’s urine is drunk | oral | [14] |
| Portugal | Dog (Canis lupus familiaris) | Urine | Wound: dog’s urine is applied to the wound | topical | [14] |
| Portugal | Human (Homo sapiens) | Urine | Heals wasp stings: children’s urine mixed with mud is applied to the afflicted area | topical | [14] |
| Portugal | Human (Homo sapiens) | Urine | Against fever: urine from a little girl | ns | [14] |
| Portugal | Human (Homo sapiens) | Urine | Heals intestinal infections: urine from a little boy is drunk | oral | [14] |
| Portugal | Human (Homo sapiens) | Urine | Against jaundice: urine from a little boy is drunk | oral | [14] |
| Portugal | Human (Homo sapiens) | Urine | Against toothache: a pulp of urine and flour is placed on the afflicted tooth | topical | [14] |
| Portugal | Human (Homo sapiens) | Urine | Against cramps: urine from a little boy is drunk | oral | [14] |
| Portugal | Human (Homo sapiens) | Urine | Against dermatophytosis: children’s urine mixed with mud is applied to the afflicted area | topical | [14] |
| Portugal | Human (Homo sapiens) | Urine | Heals wounds: human urine is applied over the wound | topical | [14] |
| Rodriguez island (Mascarene archipelago) | Sheep (Ovis aries) | Faeces | Expextorant, cough, asthma: an infusion of the dried sheep dung is prepared and 1 cup is administered orally once. For asthma sometime fresh sheep milk is added. | oral | [42] |
| Rodriguez island (Mascarene archipelago) | Sheep (Ovis aries) | Faeces | Asthma : an infusion of the dried sheep dung is prepared and 1 cup is administered orally once. (in one UR for asthma milk is adde to infusion) | oral | [42] |
| Saudi Arabia and Jordan | Arabian sand gazelle (Gazella marica) | Faeces | Strengthen the growth of hair: faeces are dissolved in water and the paste created is scrubbed on body | topical | [3] |
| Saudi Arabia and Jordan | Rock Hyrax (Procavia capensis) | Faeces | Expectorant treatment for sever cough, faeces mixed with honey and fenugrec | ns | [3] |
| Sikkhim | Bos taurus | Urine | Diabetes: Urine is collected, some water is added and then immediately given to the patient for consumption | oral | [20] |
| South Africa | Cow (Bos taurus) | Faeces | Diarrhoea, fever and ache, skin infection, anti-toxin | ns | [47] |
| South Africa | Cow (Bos taurus) | Urine | Diarrhoea, fever and ache, Throat Infection, haemorrhoid, skin infection, anti-toxin | ns | [47] |
| Spain | Dog (Canis lupus familiaris) | Faeces | Reconstituent for malnourished children: dog faeces are exposed to open air overnight and then boiled. This water is filtered and given to drink | oral | [51] |
| Spain | Dog (Canis lupus familiaris) | Faeces | anti-rhinitic: dog feces are prepared in a tea with dry figs (Ficus carica), ears of corn (Zea mays), snake skin and honey | oral | [51] |
| Spain | Donkey (Equus asinus) | Faeces | Promote placenta expulsion: donkey’s feces is boiled and the liquid is drunk by women after giving birth | oral | [51] |
| Spain | RockPigeon (Columba livia, wood pigeon (C.palomas), stock pigeons (C.oenas) | Faeces | Ingrown nail, acne: crumbs and applied | topical | [51] |
| Spain | Common pigeon (Columba livia) | Faeces | Antipneumonic: poultice | topical | [53] |
| Spain | Dog (Canis lupus familiaris) | Faeces | Antipneumonic, antidiphteric, sore throat: poultice | topical | [53] |
| Spain | Hen (Gallus domesticus) | Faeces | Antipneumonic: poultice | topical | [53] |
| Spain | Dog (Canis lupus familiaris) | Faeces | Diarrhoea: boiled (Internal use) | oral | [57] |
| Spain | Dog (Canis lupus familiaris) | Faeces | Nose bleeding: nose is blocked with dog faeces | topical | [57] |
| Spain | Dog (Canis lupus familiaris) | Faeces | Malnutrition: dog faeces were left in the open air overnight and then boiled. This water was filtered and given to malnourished children to drink, as a reconstituent tonic. | oral | [57] |
| Spain | Dog (Canis lupus familiaris) | Faeces | Diarrhoea: dried dog faeces are cooked and the resulting broth is drunk | oral | [57] |
| Spain | Dog (Canis lupus familiaris) | Faeces | Nose bleed: applied in the nose without preparation | topical | [57] |
| Spain | Dog (Canis lupus familiaris) | Faeces | Colds: Tisane with different components is drunk | oral | [57] |
| Spain | Human (Homo sapiens) | Urine | Emollient, analgesic–antinflammatory topical application | topical | [10] |
| Spain | Human (Homo sapiens) | Urine | Emollient for chapped hands: urine is used to wash the hands | topical | [51] |
| Sudan | Crested porcupine (Hystrix cristata) | Faeces | Jaundice, Diabetes: Feces soaked in water and taken orally | oral | [1] |
| Sudan | Domestic rabbit (cuniculus Oryctolagus) | Faeces | Abdominal colic especially for infants: faeces filtered drink | oral | [1] |
| Sudan | Grasshoppers | Faeces | Abdominal colic : faeces soaked in water and taken orally | oral | [1] |
| Sudan | Hedgehog (Hemiechinus aethiopicus) | Faeces | Trachoma: feces taken orally | ns | [1] |
| Sudan | One humped camel (Camelus dromedariu) | Faeces | Tinea capitis: feces mixed with latex of Clatopris procera | ns | [1] |
| Sudan | Alligator (Varanus niloticus) | Faeces | An infusion prepared from the dung is dropped on the eyes to improve eyesight | topical | [23] |
| Sudan | Camel (Camelus dromedarius) | Faeces | The fresh dung is applied externally on the infected parts to relieve arthritis. | topical | [23] |
| Sudan | Crocodile (Crocodylus niloticus) | Faeces | An infusion prepared from the dung is dropped on the eyes to improve eyesight. | topical | [23] |
| Sudan | One humped camel (Camelus dromedariu) | Urine | urine of a nursing mother: whopping cough urine: antiseptic for wound healing | ns | [1] |
| Sudan | Rock hyrax (Procavia capensis) | Urine | Ulcers infections, heart problems, promote immunity | ns | [1] |
| Sudan | Camel (Camelus dromedarius) | Urine | The urine placed in a copper container is dropped on eyes to improve eyesight. | topical | [23] |
| Sudan | Dog (Canis lupus familiaris) | Urine | The urine is applied externally on skin to treat warts. | topical | [23] |
| Tanzania | African elephant | Faeces | Fumigation with smoke from elephant dung in case of child convulsions *(degedege)* | other | [65] |
| Tanzania | Rock Hyrax (Procavia capensis) | Urine | Syphilis Collect hyrax urinated soil; mix water; filter soil and then drink | oral | [58] |
| Thailand | Human (Homo sapiens) | Urine | Ethnoveterinary use: applied to eye cataract (for elephants) | Topical | [27] |
| Zimbabwe | African elephant | Faeces | Maceration drunk from 3rd trimester [of pregnancy] to facilitate birth and to prevent tears | oral | [66] |
| Zimbabwe | African elephant | Faeces | To quicken labour: elephant dung is soaked in water and drunk at the onset of labour | oral | [67] |

References :

1. Adam SA, Aldow BM, Mahmoud ZN, Saad AA, Mahmoud S, Mahmoud SZ, et al. Studies on Ethnozoology in Sudan: 1. Zootherapeutic Practices. 2020;3:6.

2. Akindele SO, Akinnifesi F, Dawson-Andoh B, Fuwape JA, Stimm B, Adedire MO. Use of Wild Animals as.Alternative Therapy in Support Zone Villages Around Some Nigeria National Parks. Forest and Forest Products Journal. 2010;5:7–18.

3. Aloufi A, Eid E. Zootherapy: A study from the Northwestern region of the Kingdom of Saudi Arabia and the Hashemite Kingdom of Jordan. 2016;15:9.

4. Alves RRN, Rosa IL. Zootherapeutic practices among fishing communities in North and Northeast Brazil: A comparison. Journal of Ethnopharmacology. 2007;111:82–103.

5. Alves R, Santana G, Almeida W, Léo Neto N, Vieira W. Reptiles used for medicinal and magic religious purposes in Brazil. Appl Herpetol. 2009;6:257–74.

6. Alves RRN, Neta RO de S, Trovão DM de BM, Barbosa JE de L, Barros AT, Dias TLP. Traditional uses of medicinal animals in the semi-arid region of northeastern Brazil. J Ethnobiology Ethnomedicine. 2012;8:41.

7. Bagde N, Jain S. An ethnozoological studies and medicinal values of vertebrate origin in the adjoining areas of Pench National Park of Chhindwara District of Madhya Pradesh, India. Ind Int J Life Sci. 2013;1:278–83.

8. Baird IG. Lao PDR: an overview of traditional medicines derived from wild animals and plants. TRAFFIC Southeast Asia. 1995;

9. Barboza RR, de MS Souto W, da S Mourão J. The use of zootherapeutics in folk veterinary medicine in the district of Cubati, Paraíba State, Brazil. J Ethnobiology Ethnomedicine. 2007;3:32.

10. Benítez G. Animals used for medicinal and magico-religious purposes in western Granada Province, Andalusia (Spain). Journal of Ethnopharmacology. 2011;137:1113–23.

11. Sajem Betlu AL. Indigenous knowledge of zootherapeutic use among the Biate tribe of Dima Hasao District, Assam, Northeastern India. Journal of Ethnobiology and Ethnomedicine. 2013;9:56.

12. Borah PK, Gogoi P, Phukan AC, Mahanta J. Traditional medicine in the treatment of gastrointestinal diseases in Upper Assam. IJTK Vol5(4) [October 2006]. 2006;

13. Braga-Pereira F, Santoro FR, Santos CV-D, Alves RRN. First record of Angola’s medicinal animals: A case study on the use of mammals in local medicine in Quiçama National Park. 2017;16:5.

14. Ceríaco LMP. A Review of Fauna Used in Zootherapeutic Remedies in Portugal: Historical Origins, Current Uses, and Implications for Conservation. In: Alves RRN, Rosa IL, editors. Animals in Traditional Folk Medicine: Implications for Conservation. Berlin, Heidelberg: Springer; 2013. p. 317–45.

15. Chassagne F, Hul S, Deharo E, Bourdy G. Natural remedies used by Bunong people in Mondulkiri province (Northeast Cambodia) with special reference to the treatment of 11 most common ailments. Journal of ethnopharmacology. 2016;191:41–70.

16. Chinlampianga M, Singh RK, Shukla AC. Ethnozoological diversity of Northeast India: Empirical learning with traditional knowledge holders of Mizoram and Arunachal Pradesh. NISCAIR-CSIR, India; 2013;

17. Costa-Neto EM. Implications and applications of folk zootherapy in the state of Bahia, Northeastern Brazil. Sust Dev. 2004;12:161–74.

18. de Melo RS, da Silva OC, Souto A, Alves RRN, Schiel N. The Role of Mammals in Local Communities Living in Conservation Areas in the Northeast of Brazil: An Ethnozoological Approach. Tropical Conservation Science. 2014;7:423–39.

19. Devi OB, Devi LR, Singh WM, Devi AR. Traditional Medicines and Health Care from the animals of Manipur, India. International Journal of Scientific and Research Publications. 2015;Volume 5, Issue 11, November 2015 Edition.

20. Dhakal P, Chettri B, Lepcha S, Acharya BK. Rich yet undocumented ethnozoological practices of socio-culturally diverse indigenous communities of Sikkim Himalaya, India. Journal of Ethnopharmacology. 2020;249:112386.

21. Dixit AK, Kadavul K, Rajalakshmi S, Shekhawat MS. Ethno-medico-biological studies of South India. IJTK Vol9(1) [January 2010]. CSIR; 2010;

22. Dubost J-M, Lamxay V, Krief S, Falshaw M, Manithip C, Deharo E. From plant selection by elephants to human and veterinary pharmacopeia of mahouts in Laos. Journal of Ethnopharmacology. 2019;244:112157.

23. El-Kamali HH. Folk medicinal use of some animal products in Central Sudan. J Ethnopharmacol. 2000;72:279–82.

24. Estrada ZJ, Panolino J, De Mesa TK, Abordo FC, Labao R. An Ethnozoological Study of the Medicinal Animals Used by the Tagbanua Tribe in Sitio Tablay, Cabigaan, Aborlan, Palawan. Science, Technology and Innovation for Sustainable Development Proceedings of the 2nd Palawan Research Symposium 2015. 2015;123.

25. Friant S, Bonwitt J, Ayambem WA, Ifebueme NM, Alobi AO, Otukpa OM, et al. Zootherapy as a Potential Pathway for Zoonotic Spillover: a Mixed-methods Study of the Use of Animal Products in Medicinal and Cultural Practices in Nigeria. In Review; 2021 May.

26. Gogoi C, Bora M. Zoo-therapeutic practices among the deori tribes of Dhemaji district, Assam, India.

27. Greene AM, Panyadee P, Inta A, Huffman MA. Asian elephant self-medication as a source of ethnoveterinary knowledge among Karen mahouts in northern Thailand. Journal of Ethnopharmacology. 2020;259:112823.

28. Gupta L, Silori CS, Mistry N, Dixit AM. Use of Animals and Animal products in traditional health care systems in District Kachchh, Gujarat. 2003;

29. Jamir NS, Lal P. Ethnozoological practices among Naga tribes. 2005;4:5.

30. Jaroli D, Mahawar MM, Vyas N. An ethnozoological study in the adjoining areas of Mount Abu wildlife sanctuary, India. J Ethnobiology Ethnomedicine. 2010;6:6.

31. Jayakumar S, Sathiskumar S, Baskaran N, Arumugam R, Vanitha V. Ethno-veterinary practices in Southern India for captive Asian elephant ailments. J Ethnopharmacol. 2017;200:182–204.

32. Jugli S, Chakravorty J, Meyer-Rochow VB. Zootherapeutic uses of animals and their parts: an important element of the traditional knowledge of the Tangsa and Wancho of eastern Arunachal Pradesh, North-East India. Environ Dev Sustain. 2019;

33. Kakati LN, Doulo V. Indigenous knowledge system of zootherapeutic use by Chakhesang tribe of Nagaland, India. Journal of Human Ecology. Taylor & Francis; 2002;13:419–23.

34. Kakati LN, Ao B, Doulo V. Indigenous Knowledge of Zootherapeutic Use of Vertebrate Origin by the Ao Tribe of Nagaland. Journal of Human Ecology. Routledge; 2006;19:163–7.

35. Kamble VS. ETNOZOOLOGICAL SURVERY OF NOMADIC TRIBS FROM DROUGHT PRONE REGION SANGOLA, DIST. SOLAPUR (MS) INDIA. antiquity. 2014;19:20.

36. Kendie FA, Mekuriaw SA, Dagnew MA. Ethnozoological study of traditional medicinal appreciation of animals and their products among the indigenous people of Metema Woreda, North-Western Ethiopia. J Ethnobiology Ethnomedicine. 2018;14:37.

37. Khan FM, Chaudhry H, Mustafa YS, Ahmad W, Farhan HM. Ethno-veterinary zoo-therapies and occult practices in greater Cholistan desert (Pakistan). Sci Int (Lahore). 2011;23:241–3.

38. Lalramnghinglova H. Ethnobiology in Mizoram state: folklore medico-zoology. Bulletin of the Indian Institute of History of Medicine (Hyderabad). 1999;29:123–48.

39. Lohanan R. The elephant situation in Thailand and a plea for co-operation. 2016.

40. Mahawar MM, Jaroli D. Animals and their products utilized as medicines by the inhabitants surrounding the Ranthambhore National Park, India. Journal of Ethnobiology and Ethnomedicine. 2006;2:46.

41. Mahawar MM, Jaroli D. Traditional knowledge on zootherapeutic uses by the Saharia tribe of Rajasthan, India. J Ethnobiology Ethnomedicine. 2007;3:25.

42. Mahomoodally F, Samoisy AK, Suroowan S. Ethnozoological practices in Rodrigues island of the Mascarene archipelago. Journal of Ethnopharmacology. 2019;245:112163.

43. Misar SD, Subhas M, Khinchi PJ, Mohitkar SP. ETHNOZOOLOGICAL STUDIES AMONG ADJOINING AREAS OF PANDHARKAWDA TALUKA OF YAVATMAL DISTRICT, MAHARASHTRA, INDIA. INTERNATIONAL JOURNAL OF RESEARCHES IN BIOSCIENCES, AGRICULTURE AND TECHNOLOGY. 2016;4.

44. MUWEL A, KHER A, SHIVANI K. PLANTS, ANIMALS AND THEIR PRODUCTS UTILIZED AS TRADITIONAL MEDICINES IN BHILALA TRIBE OF DISTRICT DHAR OF MADHYAPRADESH INDIA-A PRELEMINARY SURVEY by ALKESH MUWEL, ARUN KHER AND KAMLA SHIVANI. LIFE SCIENCES LEAFLETS. 2016;79:65-to.

45. Negi CS, Palyal VS. Traditional Uses of Animal and Animal Products in Medicine and Rituals by the Shoka Tribes of District Pithoragarh, Uttaranchal, India. Studies on Ethno-Medicine. Routledge; 2007;1:47–54.

46. Negi T, Kandari LS. Traditional knowledge and zootherapeutic use of different animals by <em>Bhotiya</em> tribe: A case study from Uttarakhand, India. IJTK Vol16(4) [October 2017] [Internet]. NISCAIR-CSIR, India; 2017 [cited 2020 Jul 26]; Available from: http://nopr.niscair.res.in/handle/123456789/42660

47. Oyedeji-Amusa MO, Ojuromi OT, Ashafa AO. Ethnoveterinary survey of tradomedical importance of Bos taurus L urine, bile and dung in Nigeria and South Africa. Tropical Journal of Pharmaceutical Research. 2016;15:1807-1813–1813.

48. Pieroni A, Quave CL, Santoro RF. Folk pharmaceutical knowledge in the territory of the Dolomiti Lucane, inland southern Italy. Journal of Ethnopharmacology. 2004;95:373–84.

49. Pieroni A, Giusti ME, Quave CL. Cross-Cultural Ethnobiology in the Western Balkans: Medical Ethnobotany and Ethnozoology Among Albanians and Serbs in the Pešter Plateau, Sandžak, South-Western Serbia. Hum Ecol. 2011;39:333.

50. Pieroni A, Ibraliu A, Abbasi AM, Papajani-Toska V. An ethnobotanical study among Albanians and Aromanians living in the Rraicë and Mokra areas of Eastern Albania. Genet Resour Crop Evol. 2015;62:477–500.

51. Quave CL, Lohani U, Verde A, Fajardo J, Rivera D, Obón C, et al. A Comparative Assessment of Zootherapeutic Remedies from Selected Areas in Albania, Italy, Spain and Nepal. Journal of Ethnobiology. 2010;30:92–125.

52. Raja L, Matheswaran P, Anbalagan M, Sureshkumar V, Ganesan D, Gani SB. Ethnozoological study of animal-based products practices among the tribal inhabitants in Kolli Hills Namakkal District, Tamil Nadu, India. World Journal of Pharmacy and Pharmaceutical Sciences. 2018;7:785–97.

53. Rigat M, Vallès J, Iglésias J, Garnatje T. Traditional and alternative natural therapeutic products used in the treatment of respiratory tract infectious diseases in the eastern Catalan Pyrenees (Iberian Peninsula). Journal of Ethnopharmacology. 2013;148:411–22.

54. Ronghang R, Teron R, Tamuli AK, Rajkhowa RC. Traditional zootherapy practiced among the Karbis of Assam (India). The Ecoscan. 2011;1:161–6.

55. Shoukat A, Khan MF, Shah GM, Tabassam S, Sajid M, Siddique H, et al. Indigenous knowledge of zootherapeutic use among the people of Hazara division Khyber-Pakhtunkhwa, Pakistan. IJTK Vol19(3) [July 2020]. NISCAIR-CSIR, India; 2020;

56. Solavan A, Paulmurugan R, Wilsanand V, Sing AJAr. Traditional therapeutic uses of animals among tribal population of Tamil Nadu. 2004;3:8.

57. Vallejo JR, González JA. THE MEDICAL USE OF LEECHES IN CONTEMPORARY SPAIN: BETWEEN SCIENCE AND TRADITION. Acta medico-historica Adriatica : AMHA. Hrvatsko znanstveno društvo za povijest zdravstvene kulture; 2015;13:131–58.

58. Vats R, Thomas S. A study on use of animals as traditional medicine by Sukuma Tribe of Busega District in North-western Tanzania. Journal of ethnobiology and ethnomedicine. Springer; 2015;11:1–11.

59. Verma AK, Prasad SB, Rongpi T, Arjun J. Traditional healing with animals (zootherapy) by the major ethnic group of Karbi Anglong district of Assam, India. International journal of Pharmacy and Pharmaceutical sciences. 2014;6:593–600.

60. Vijayakumar S, Prabhu S, Morvin Yabesh JE, Pragashraj R. A quantitative ethnozoological study of traditionally used animals in Pachamalai hills of Tamil Nadu, India. Journal of Ethnopharmacology. 2015;171:51–63.

61. Volpato G, Lamin Saleh SM, Di Nardo A. Ethnoveterinary of Sahrawi pastoralists of Western Sahara: camel diseases and remedies. Journal of Ethnobiology and Ethnomedicine. 2015;11:54.

62. Vyas N, Mahawar MM, Jaroli DP. Traditional Medicines Derived from Domestic Animals Used by Rebari Community of Rajasthan, India. 1. 2009;7:129–38.

63. Yirga G, Teferi M, Gebreslassea Y. Ethnozoological study of traditional medicinal animals used by the people of Kafta-Humera District, Northern Ethiopia. International Journal of Medicine and Medical Sciences. 2011;3:316–20.

64. Padmanabhan P, Sujana KA. Animal products in traditional medicine from Attappady hills of Western Ghats. IJTK Vol7(2) [April 2008]. CSIR; 2008;

65. Comoro C, Nsimba SED, Warsame M, Tomson G. Local understanding, perceptions and reported practices of mothers/guardians and health workers on childhood malaria in a Tanzanian district—implications for malaria control. Acta Tropica. 2003;87:305–13.

66. Mawoza T, Nhachi C, Magwali T. Prevalence of Traditional Medicine Use during Pregnancy, at Labour and for Postpartum Care in a Rural Area in Zimbabwe. Clin Mother Child Health. 2019;16:321.

67. Panganai T, Shumba P. The African Pitocin - a midwife’s dilemma: the perception of women on the use of herbs in pregnancy and labour in Zimbabwe, Gweru. Pan Afr Med J. 2016;25:9.

68. Kebebew M, Mohamed E, Meyer-Rochow VB. Knowledge and Use of Traditional Medicinal Animals in the Arba Minch Zuriya District, Gamo Zone, Southern Ethiopia. European Journal of Therapeutics. AVES; 2021;27:158–68.
