# Supplementary material for: Zootherapeutic uses of animals excreta: the case of elephant dung and urine use in Sayaboury province, Laos
Source: J Ethnobiol Ethnomed. 2021 Oct 28;17:62. doi: 10.1186/s13002-021-00484-7 (PMC8552211; doi:10.1186/s13002-021-00484-7)
Supplement: Supplementary file 2 — Additional file 2. Inventory of plants consumed by elephants. [file 13002_2021_484_MOESM2_ESM.docx]

**Appendix 2**

Inventory of plants consumed by elephants with mention of their use or not by traditional healers

| **Voucher** | **Family** | **Botanical name** | **L.F** | **Local name** | **Roman**  **transcription** | **Use by TH** |
| --- | --- | --- | --- | --- | --- | --- |
| JMD 1110 | Amarantaceae | Amaranthus spinosus L. | H | ຜັກ​ຫົມ​ໜາມ | phak hom nam | y |
| JMD 1121 | Anacardiaceae | Allospondias lakonensis (Pierre) Stapf | T | ໄມ້ສົ້ມ​ຫໍ້ | mai som ho | y |
| JMD 925 | Anacardiaceae | Gluta cambodiana Pierre | T | ໄມ້ຮັກ​ຟ້າ | mai hak fa | n |
| JMD 1099 | Anacardiaceae | Mangifera caloneura Kurz | T | ໄມ້ມ່ວງ​ປ່າ | mai muang pa | n |
| JMD 1115 | Anacardiaceae | Spondias pinnata (L.f.) Kurz | T | ໄມ້ໝາກກອກ / ໄມ້ກອກ | mai mak kok / mai kok | y |
| JMD 1008 | Annonaceae | Anomianthus dulcis (Dunal) J.Sinclair | T | ໄມ້ແຮກ | mai haek | y |
| JMD 1026 | Annonaceae | Goniothalamus laoticus (Finet & Gagnep.) Bân | T | ໄມ້ຍາບກະແດ້ງ | mai nyap kadeang | y |
| JMD 1111 | Apocynaceae | Amalocalyx microlobus Pierre ex Spire | C | ເຄືອຫມາກຊີມ | khuea mak sim | y |
| JMD 961 | Apocynaceae | Holarrhena pubescens Wall. ex G.Don | T | ໄມ້ເຂົາຂິວ / ໄມ້ກະແຊງ | mai khao khio / mai kasaeng | n |
| JMD 1017 | Apocynaceae | Wrightia arborea (Dennst.) Mabb. | T | ໄມ້ສະມອກ | mai samok | n |
| JMD 1031 | Araceae | Lasia spinosa (L.) Thwaites | H | ຫົວຜັກໜາມ / ບອນໜາມ | hua phak nam / bon nam | y |
| JMD 957 | Araliaceae | Heteropanax fragrans (Roxb.) Seem. | T | ໄມ້ອ້ອຍຊ້າງ / ໄມ້ຊ້າງເນົ່າ | mai oy sang / mai sang nao | y |
| JMD 908 | Araliaceae | Trevesia palmata (Lindl.) Vis. | T | ໄມ້ດອກຕ້າງ | mai dok tang | n |
| JMD 1067 | Asparagaceae | Dracaena fragrans (L.) Ker Gawl. | H | ປາລະມີ | palami | n |
| JMD 1071 | Asparagaceae | Dracaena angustifolia (Medik.) Roxb. | Sh | ​ໄມ້ບີ​ກະ​ທິງ | mai bi kathing | y |
| JMD 1119 | Asteraceae | Crassocephalum crepidioides (Benth.) S.Moore | H | ຫຍ້າຄໍເງາະ / ຫຍ້າເຮືອບີນ / ຍົນ​ເຫາະ | nya kho ngo / nya huea bin / nyon ho | y |
| JMD 1059 | Bignoniaceae | Oroxylum indicum (L.) Kurz | T | ໄມ້ໝາກລີ່ນ | mai mak lin | y |
| JMD 1058 | Burseraceae | Canarium subulatum Guillaumin | T | ໄມ້ກອກ​ກັນ | mai kok kan | y |
| JMD 1004 | Burseraceae | toona sp. | T | ໄມ້ສະຄ້ຳ | mai sakham | y |
| JMD 1108 | Combretaceae | Terminalia mucronata Craib & Hutch. | T | ໄມ້ເປືອຍເລືອດ | mai pueay lueat | y |
| JMD 1081 | Cyperaceae | Scleria ciliaris Nees. | H | ຫຍ້າສາມຫຼຽມ | nya sam liam | y |
| JMD 1091 | Dilleniaceae | Dillenia aurea Sm. | T | ໄມ້ສ້ານ​ຊ້າງ / ສ້ານ / ສ້ານ​ແພງ | mai san sang / san / san phaeng | n |
| JMD 1079 | Dilleniaceae | Dillenia indica L. | T | ໄມ້ສ້ານ​ໝາກ​ໃຫ​ຍ່ | mai san mak nyai | y |
| JMD 1010 | Dilleniaceae | Dillenia obovata (Blume) Hoogland | T | ໄມ້ສ້ານ | mai san | n |
| JMD 1038 | Dilleniaceae | Dillenia parviflora Griff. | T | ໄມ້ສັນແຄງ | mai san khaeng | n |
| JMD 1148 | Dipterocarpaceae | Shorea siamensis (Miq.) Kurz | T | ໄມ້ຮັງ | mai hang | y |
| JMD 1007 | Euphorbiaceae | Phyllanthus sp. | C | ຂາມເຄືອ | kham khuea | y |
| JMD 1130 | Euphorbiaceae | Bischofia javanica Blume | T | ໄມ້ຂົມຝາດ | mai khom fat | y |
| JMD 962 | Euphorbiaceae | Homonoia riparia Lour. | T | ໄມ້ໄຄ້ນໍ້າ | mai khai nam | y |
| JMD 1044 | Euphorbiaceae | Mallotus barbatus Müll.Arg. | T | ໄມ້ກະເທົ້າ | mai kathao | y |
| JMD 1019 | Euphorbiaceae | ni | T | ໄມ້ໝາກເຂືອເຖື່ອນ | mai mak khuea thuean | y |
| JMD 1142 | Euphorbiaceae | Phyllanthus emblica L. | T | ໄມ້ຂາມ​ປ້ອມ | mai kham pom | y |
| JMD 995 | Euphorbiaceae | Phyllanthus reticulatus Poir. | T | ໄມ້ຮັງປ່າ | mai hang pa | y |
| JMD 1127 | Fabaceae | Acacia caesia (L.) Willd. | C | ເຄືອຫັນ | khuea han | y |
| JMD 1149 | Fabaceae | Acacia concinna (Willd.) A.DC. | C | ສົ້ມປ່ອຍ | som poi | y |
| JMD 1140 | Fabaceae | Acacia pennata (L.) Willd. | C | ເຄືອຜັກເນົ່າ | khuea phak nao | y |
| JMD 1055 | Fabaceae | Dalbergia rimosa Roxb. | C | ໄມ້ດູ່ເຄືອ | mai du khuea | y |
| JMD 1124 /1106 | Fabaceae | Entada glandulosa Pierre ex Gagnep. / Entada rheedii Spreng | C | ເຄືອແລະ | khuea lae | y |
| JMD 1112 | Fabaceae | Mucuna pruriens (L.) DC. | C | ເຄືອຕຸ່ມແຍ / ເຄືອຕຳ​ແຍ | khuea tum nyae / khuea tam nyae | y |
| JMD 1035 | Fabaceae | Pueraria montana var. lobata (Willd.) Sanjappa & Pradeep. | C | ເຄືອເຂົາຂົນ / ເຄືອໝາກ | khuea khao khon / khuea mak | y |
| JMD 1128 | Fabaceae | Spatholobus parviflorus (DC.) Kuntze | C | ເຄືອຈານ / ເຄືອຄູ້ | khuea chan / khuea khu | y |
| JMD 1030 | Fabaceae | Mimosa pudica L. | H | ຫຍ້າຍຸບແຍບ | nya nyup nyaep | y |
| JMD 1123 | Fabaceae | Albizia lucidior (Steud.) I.C.Nielsen | T | ໄມ້ສະແຄ້ / ໄມ້ສະແຂ່ | mai sakhae | n |
| JMD 1021 | Fabaceae | Bauhinia malabarica Roxb. | T | ໄມ້ສ້ຽວ | mai siao | n |
| JMD 1103 | Fabaceae | cf. Dalbergia oliveri Gamble | T | ໄມ້ຄຳ​ພີ​ເຫລືອງ | mai khamphi lueang | n |
| JMD 1012 | Fabaceae | Desmodium sp. | T | ໄມ້ຮົ່ວແຮເຫືອດ / ຖົ່ວແຮປ່າ | mai hua hae hueat / thua hae pa | y |
| JMD 996 | Fabaceae | Leucaena leucocephala (Lamk.) de Wit | T | ຫຍ້າກັນຖິນ | nya kanthin | y |
| JMD 1056 | Fabaceae | ni | T | ໄມ້ຂາມປ່າ / ໄມ້ໝາກຂາມ | mai kham pa / mai mak kham | n |
| JMD 1068 | Fabaceae | Pterocarpus macrocarpus Kurz | T | ໄມ້ດູ່ | mai du | y |
| JMD 1151 | Fagaceae | Castanopsis indica (Roxb. ex Lindl.) A.DC. | T | ໄມ້ກໍ່​ໜາມ | mai ko nam | n |
| JMD 1150 | Fagaceae | Lithocarpus auriculatus (Hickel & A.Camus) Barnett | T | ໄມ້ກໍ່​ກ້ຽງ | mai ko kiang | n |
| JMD 1104 | Fagaceae | Quercus kingiana Craib | T | ໄມ້ກໍ່ຢ່ອງ | mai ko yong | n |
| JMD 1096 | Guttiferae | Cratoxylum formosum (Jack) Dyer | T | ໄມ້ຕິ້ວນ້ອຍ | mai tio noi | n |
| JMD 1136 | Hypoxidaceae | Molineria capitulata (Lour.) Herb. | H | ​ຫຍ້າຕອງ​ເຮືອ | nya tong huea | n |
| JMD 1094 | Irvingiaceae | Irvingia malayana Oliv. | T | ໄມ້ກະ​ບົກ | mai kabok | y |
| JMD 1011 | Lecythidaceae | Careya sphaerica Roxburgh from checklist | T | ໄມ້ກະໂດນ | mai kadon | y |
| JMD 1122 | Marantaceae | Phrynium pubinerve Blume | H | ຕອງເຂົ້າຕົ້ມ | tong khao tom | n |
| JMD 1138 | Marantaceae | Stachyphrynium placentarium (Lour.) Clausager & Borchs. | H | ຕອງຈິງ | tong ching | y |
| JMD 1126 | Menispermaceae | Tinospora crispa (L.) Hook.f. & Thomson | C | ເຄືອເຂົາຮໍ | khuea khao ho | y |
| JMD 969 | Moraceae | Ficus heterophylla L. f. | C | ເຄືອນອດນຳ | khuea not nam | y |
| JMD 972 | Moraceae | Maclura cochinchinensis (Lour.) Corner | Sh | ໄມ້ໜາມແພ່ງ / | mai nam phaeng | n |
| JMD 1093 | Moraceae | Broussonetia papyrifera (L.) L’Hér. ex Vent. | T | ໄມ້ສາ | mai sa | y |
| JMD 1134 | Moraceae | Ficus hispida L.f. | T | ໄມ້ເດື່ອ​ປ່ອງ | mai duea pong | y |
| JMD 1102 | Moraceae | Ficus microcarpa L.f. | T | ໄມ້ໄຮ | mai hai | n |
| JMD 1002 | Moraceae | Ficus racemosa L. | T | ໄມ້ເດື່ອ | mai duea | y |
| JMD 1025 | Moraceae | Ficus religiosa L. | T | ໄມ້ໂພ | mai pho | y |
| JMD 1092 | Moraceae | Ficus semicordata Buch.-Ham. ex Sm. | T | ໄມ້ໝາກນອດດິນ / ໄມ້ໜາກນອດປ່າ | mai mak not din / mai nak not pa | n |
| JMD 997 | Moraceae | Streblus asper Lour. | T | ໄມ້ສົມພໍ | mai sompho | n |
| JMD 955 | Moraceae | Streblus sp. | T | ໄມ້ໜັງຄວາຍເຖົ້າ | mai nang khuay thao | n |
| JMD 903 | Palmae | Arenga pinnata (Wurmb) Merr. | P | ​ໄມ້ຕາວ / ຕາວຮ້າງ | mai tao / tao hang | y |
| JMD 954 | Palmae | Calamus rhabdocladus Burret | P | ເຄືອບຸ່ນ | khuea bun | n |
| JMD 1125 | Palmae | Calamus solitarius T. Evans et al. | P | ເຄືອຫວາຍ | khuea vai | n |
| JMD 976 | Poaceae | Bambusa bambos (L.) Voss | B | ໄມ້ໃຜ່ໜາມ | mai phai nam | y |
| JMD 935 | Poaceae | Bambusa tulda Roxb. | B | ໄມ້ບົງ | mai bong | y |
| JMD 921 | Poaceae | Cephalostachyum pergracile Munro | B | ໄມ້ເຂົ້າຫຼາມ | mai khao lam | n |
| JMD 977 | Poaceae | Dendrocalamus brandisii (Munro) Kurz | B | ໄມ້ຫົກ | mai hok | n |
| JMD 934 | Poaceae | Dendrocalamus menbranaceus Munro | B | ໄມ້ຊາງ | mai sang | y |
| JMD 927 | Poaceae | Gigantochloa albociliata (Munro) Kurz | B | ໄມ້ໄລ່ | mai lai | n |
| JMD 951 | Poaceae | Pseudostachyum polymorphum Munro | B | ໄມ້ຊອດ | mai sot | n |
| JMD 906 | Poaceae | Schizostachyum blumei Nees | B | ໄມ້ເຮ່ຍ | mai hia | y |
| JMD 990 | Poaceae | Thyrsostachys siamensis Gamble | B | ໄມ້ຮວກ | mai huak | y |
| JMD 1129 | Poaceae | Centotheca lappacea (L.) Desv. | H | ຫຍ້າລີ​ແພ | nya li phae | y |
| JMD 1117 | Poaceae | Imperata cylindrica (L.) Raeusch. | H | ຫຍ້າຄາ | nya kha | y |
| JMD 1085 | Poaceae | Microstegium ciliatum (Trin.) A.Camus | H | ຫຍ້າເຄືອ | nya khuea | y |
| JMD 1062 | Poaceae | ni | H | ຫຍ້າປ່ອງ / ຫຍ້າຂົມບາງ | nya bong / nya khom bang | y |
| JMD 932 | Poaceae | Pennisetum polystachion (L.) Schult. | H | ຫຍ້າຂະໜົງ | nya khanong | n |
| JMD 993 | Poaceae | Phragmites australis (Cav.) Trin. ex Steud. | H | ຫຍ້າອອ້ຍຫນູ | nya oi nu | y |
| JMD 1045 | Poaceae | Saccharum arundinaceum L. | H | ຫຍ້າເລົ່າ | nya lao | n |
| JMD 911 | Poaceae | Thysanolaena latifolia (Hornem.) Honda | H | ຫຍ້າແຂມ | nya khaem | y |
| JMD 994 | Poaceae | Rubus pluribracteatus L.T.Lu & Boufford. | Sh | ໄມ້ໝາກນອດນ້ຳ | mai mak not nam | n |
| JMD 1023 | Rhamnaceae | Ziziphus jujuba Mill. | C | ກຳ​ລັງເສືອໂຂ່ງ | kamlang suea khong | y |
| JMD 1100 | Rubiaceae | Gardenia sootepensis Hutch. | T | ໄມ້ດອກໝອກ / ຕົ້້ນໄຂເນົ່າ | mai dok mok / mai khai nao | y |
| JMD 1109 | Rubiaceae | Hymenodictyon orixense (Roxb.) Mabb. | T | ໄມ້ສົ້ມ​ກົບ | mai som kop | y |
| JMD 915 | Rubiaceae | Neonauclea purpurea (Roxb.) Merr. | T | ໄມ້ສະໂກ້ | mai sako | y |
| JMD 1049 | Rubiaceae | Paederia foetida L. | T | ເຄືອ​ຕົດ​ໝາ | khuea tot ma | y |
| JMD 1051 | Sapindaceae | Lepisanthes rubiginosa (Roxb.) Leenh. | T | ໄມ້ໝາກຮວດ | mai mak huat | y |
| JMD1144 | Simaroubaceae | Harrisonia perforata (Blanco) Merr. | Sh | ໄມ້ຫນາມເຈຍ | mai nam chia | y |
| JMD 1139 | Stemonaceae | Stemona tuberosa Lour. | H | ຫົວສາມສິບ | hua sam sip | n |
| JMD 958 | Theaceae | Pyrenaria sp. | T | ໄມ້ຕັບເຕົ່າ / ໄມ້ເນົ່າໃນ | mai tap tao / mai nao nai | y |
| JMD 1064 | Thymelaeaceae | Aquilaria sp. | T | ໄມ້ຫຍ້າຮາກດຽວ | mai nya hak diao | y |
| JMD 1145 | Tiliaceae | Colona floribunda (Kurz) Craib | T | ໄມ້ປໍແດງ | mai po daeng | n |
| JMD 1105 | Tiliaceae | Colona merguensis (Planch. ex Mast.) Burret | T | ໄມ້ຫຍາບ​ປາ​ຊິວ | mai nyap pa sio | n |
| JMD 1029 | Ulmaceae | Trema orientalis (L.) Blume | T | ໄມ້ສາສ້ອຍ | mai sa soi | y |
| JMD 1090 | Verbenaceae | Vitex pinata L. | T | ໄມ້ຕິີນ​ເປັດ | mai tin pet | y |
| JMD 1048 | Verbenaceae | Vitex trifolia L. | T | ໄມ້ຕີນນົກ | mai tin nok | n |
| JMD 1131 | Vitaceae | Ampelocissus martini Planch. | C | ເຄືອເຂົາດີນ | khuea khao din | y |
| JMD 1013 | Vitaceae | Ampelocissus sp. | C | ເຄືອສົ້ມລົມ | khuea som lom | y |
| JMD 1053 | Vitaceae | ni | C | ເຄືອດອກຈໍ້ | khuea dok cho | n |
| JMD 933 | Zingiberaceae | Alpinia galanga (L.) Willd. | H | ຂ່າ | kha | y |
| JMD 1047 | Zingiberaceae | Alpinia sp. | H | ຂ່າໂຄມ | kha khom | y |
| JMD 1113 | Zingiberaceae | Amomum schmidtii (K.Schum.) Gagnep. | H | ຫຍ້າແໜ່ງ | nya naeng | y |
| JMD 1137 | Zingiberaceae | Amomum villosum Lour. | H | ແໜ່ງ​ຂຽວ | naeng khiao | n |
| JMD 1089 | Zingiberaceae | Ellingera sp. | H | ຫຍ້າຄ່າ​ໂຄມ / ໝາກ​ເຕີ | nya kha khom / mak toe | y |

(col. 4 : LF = Life Form, B = bamboo, C = climber, H = herb, Sh = shrub, T = Tree. col 7: TH = Traditional healer, y = yes, n = no)
